# Association of *LDLR* and *SCARB1* gene polymorphisms with hepatocellular carcinoma: a case-control study proposing a ‘double-hit’ model

**DOI:** 10.1186/s12944-026-02946-x

**Published:** 2026-04-11

**Authors:** Zuhal Altintas, Muammer Ozgur Cevik, Ebru Derici Eker, Engin Altintas

**Affiliations:** 1 Department of Medical Genetics, Faculty of Medicine, Mersin University, Mersin, 33310 Türkiye; 2 Department of Medical Genetics, Faculty of Medicine, Adiyaman University, Adiyaman, 02040 Türkiye; 3 Department of Pharmaceutical Technology, Faculty of Pharmacy, Mersin University, Mersin, 33169 Türkiye; 4 Department of Gastroenterology, Faculty of Medicine, Mersin University, Mersin, 33310 Türkiye

## Abstract

**Background:**

Low-density lipoprotein receptor (*LDLR*) and scavenger receptor class B member 1 (*SCARB1*) are essential regulators of systemic lipid homeostasis. Although dysregulated lipid metabolism is a hallmark of malignancy, the genetic associations between these receptors and the risks of hepatocellular carcinoma (HCC) remain poorly understood. This study aimed to evaluate the impacts of *LDLR* and *SCARB1* polymorphisms on the susceptibility to HCC and clinical biochemical profiles in a population with a high prevalence of viral hepatitis.

**Methods:**

In this case‒control study, 81 HCC patients and 162 healthy controls were genotyped for the *SCARB1* rs4238001 and *LDLR* rs688 and rs5925 variants by using an automated capillary electrophoresis system. The correlations between the genetic data and clinical markers of liver injury, including alanine aminotransferase (ALT) and aspartate aminotransferase (AST) levels, as well as low-density lipoprotein cholesterol (LDL-C) levels, were evaluated. Statistical analyses used logistic regression for risk estimation and nonparametric tests (medians and interquartile ranges) for biochemical correlations.

**Results:**

The *LDLR* rs5925 polymorphism emerged as a major risk factor for HCC. According to the recessive model, compared with the reference genotypes, the homozygous CC genotype was associated with a 13.05-fold greater risk of HCC [Odds ratios (OR) = 13.05, 95% CI: 3.69–46.12; *P* < 0.001]. According to the dominant model, carriers of the minor C allele had a 6.94-fold increased risk of HCC (OR = 6.94, 95% CI: 3.82–12.60; *P* < 0.001). Additionally, the *LDLR* rs688 variant was linked to metabolic alterations, with the TT homozygotes having significantly higher LDL-C levels (*P* = 0.025) and the CT heterozygotes having elevated ALT levels (*P* = 0.002). Notably, the rs5925 CC genotype strongly correlated with elevated AST levels (*P* = 0.022), which suggested a synergistic link between genetic predisposition and hepatocellular injury. Building on these findings, we conducted an interaction analysis, which supported an integrated ‘double-hit’ model, demonstrating for the first time how the synergy between *LDLR* and *SCARB1* polymorphisms modulates the HCC risk through disrupted lipid homeostasis.

**Conclusion:**

The findings demonstrate that the *LDLR* rs5925 variant is a potent genetic marker for susceptibility to HCC. The association of *LDLR* variants with impaired lipid homeostasis and hepatic stress supports a ‘double-hit’ model in which genetic vulnerability and chronic viral hepatitis factors synergistically promote hepatocarcinogenesis. These results highlight the potential utility of genetic screening for personalized risk stratification of patients with chronic liver disease.

**Supplementary Information:**

The online version contains supplementary material available at 10.1186/s12944-026-02946-x.

## Introduction

Hepatocellular carcinoma (HCC) is the predominant primary liver malignancy and ranks as the second leading cause of cancer-related mortality worldwide [1, 2]. The global incidence of HCC is steadily increasing, particularly in regions with a high prevalence of chronic viral hepatitis, such as hepatitis B virus (HBV) and hepatitis C virus (HCV) infections. While established risk factors, including viral hepatitis, chronic alcohol consumption, metabolic syndrome, obesity, and nonalcoholic fatty liver disease (NAFLD), drive the majority of cases, emerging evidence highlights the critical role of lipid metabolism dysregulation in hepatocarcinogenesis [1, 3, 4].

As the central hub for cholesterol synthesis and metabolism, the liver maintains systemic lipid homeostasis. Cholesterol is not only a vital structural component of cell membranes but also a precursor for signalling molecules. In HCC, the metabolic landscape is frequently reprogrammed, with tumour cells often exhibiting decreased cholesterol excretion, whereas its uptake and endogenous production are accelerated. This metabolic shift fosters a proinflammatory environment, induces lipotoxicity, and promotes hepatic fibrosis, all of which are precursors to malignant transformation [4, 5].

*LDLR* is a key modulator of the endocytosis of cholesterol-rich LDL particles from the systemic circulation. Polymorphisms within the *LDLR* gene can significantly impair receptor efficiency, leading to abnormal lipid accumulation in the liver and elevated systemic levels of lipids [4]. Experimental models have established that *LDLR* dysfunction serves as a critical catalyst for hepatocarcinogenesis by triggering intracellular cholesterol sequestration and chronic oxidative stress. This metabolic disruption subsequently activates oncogenic cascades, most notably the MEK/ERK signalling pathway, thereby establishing a mechanistic link between systemic lipid disorders and the molecular initiation of tumorigenesis [6]. Similarly, the scavenger receptor class B member 1 (*SCARB1*) gene facilitates selective cholesterol uptake and efflux. The dysregulation of *SCARB1* has been implicated in both HCC tumour progression and the replication cycles of HBV and HCV [7–9].

Despite the established role of lipid metabolism in hepatic health, the collective impact of *LDLR* and *SCARB1* genetic variations on HCC risk, particularly in populations with high viral hepatitis prevalence, remains poorly characterized. Most existing studies have focused on single-variant associations and often overlook the complex interplay between metabolic genetic predispositions and viral aetiological factors [4, 8, 10, 11]. Therefore, this study was designed to evaluate the synergistic influence of *LDLR* and *SCARB1* polymorphisms on the susceptibility to HCC and clinical biochemical profiles of patients. The central hypothesis is that genetic variants of these genes do not act in isolation but rather function through a ‘double-hit’ mechanism in which impaired lipid homeostasis and chronic viral inflammation converge to accelerate hepatocarcinogenesis. By integrating genomic data with real-world clinical parameters, the current research aimed to provide a novel multidimensional framework for personalized risk stratification in patients with chronic liver disease.

## Materials and methods

### Study population and ethical statement

This case‒control study was designed to investigate the associations between *LDLR* and *SCARB1* gene polymorphisms and the risks of developing HCC. The study population comprised 81 patients who had been diagnosed with HCC and 162 age- and sex-matched healthy controls. All participants were recruited from the Department of Gastroenterology at Mersin University between 2023 and 2024.

The HCC group comprised individuals over 18 years of age with a diagnosis established in accordance with the European Association for the Study of the Liver (EASL) guidelines [12]. Multiphasic computed tomography (CT), magnetic resonance imaging (MRI), and clinical or histological confirmation were performed where indicated, and the patients were monitored between 2023 and 2024. The control group consisted of healthy individuals with no prior history of malignancy, metabolic disorders, or chronic liver disease and served as a baseline for genetic and biochemical comparisons.

The study was conducted in strict accordance with the ethical principles of the Declaration of Helsinki [13]. The research was initiated following formal approval from the local Ethics Committee on 15 November 2023 (Decision No.: 2023/781). This primary approval covered the initial participant recruitment and the sample collection period between 2023 and 2024. To ensure comprehensive coverage of advanced genetic evaluations and the specific ‘double-hit’ interaction model analyses, an administrative update and extension to the protocol were finalized in 2025 (Ref. No.: 2025/529). This sequential approval process ensured that all stages of the investigation, from recruitment to specialized genetic data processing, were performed under rigorous institutional oversight.

### Biochemical and clinical data collection

Demographic information, liver function test (ALT, AST, ALP, and GGT) results, and complete blood counts were extracted from the hospital’s electronic medical records system for all participants. All clinical measurements were performed by using standardized laboratory techniques before the initiation of any therapeutic interventions to ensure baseline consistency. In accordance with the distribution of the biochemical data, values are reported as medians and interquartile ranges (IQRs) to maintain statistical accuracy during comparisons.

### DNA isolation and genotyping

Whole blood samples were collected into vacuum tubes containing ethylenediaminetetraacetic acid (EDTA) as an anticoagulant and stored at -20 °C until analysis. Genomic DNA extraction was performed with the PureLink genomic DNA isolation kit (Invitrogen, Carlsbad, CA, USA), which is based on the spin column method, following the manufacturer’s instructions. The quantity and purity of the extracted DNA were validated via spectrophotometric analysis (Applied Biosystems, Foster City, CA, USA).

Analysis of the *SCARB1* rs4238001 and *LDLR* rs688, rs5925, and rs5742911 genetic variants was performed via the polymerase chain reaction–restriction fragment length polymorphism (PCR–RFLP) technique. Following restriction enzyme digestion, the resulting DNA fragments were analysed by using the QIAxcel advanced system (Qiagen, Hilden, Germany), an automated capillary electrophoresis platform. This system provides high-resolution fragment separation and precise genotype calling through digital data analysis, ensuring significantly higher accuracy and reproducibility than traditional manual agarose gel electrophoresis does.

### Statistical analysis

Statistical analyses were conducted by using IBM SPSS Statistics version 29.0 and Python 3.11. The normality of the data distribution was evaluated via the Kolmogorov–Smirnov test. Descriptive statistics for continuous variables are expressed as the mean ± standard deviation or median IQR, whereas categorical variables are expressed as frequencies and percentages.

For continuous data, the Kruskal–Wallis test and Mann–Whitney U test were used for nonparametric comparisons. Genotype‒phenotype relationships and the distribution of allele frequencies were evaluated with the Pearson chi-square (χ^2^) test. Odds ratios (ORs) and 95% confidence intervals (CIs) were calculated by using a binary logistic regression model adjusted for age and sex as potential confounders. The adherence of genotype frequencies to the Hardy‒Weinberg equilibrium (HWE) was tested in the control group.

Following the reviewer’s suggestions, an a priori power analysis was performed, which showed that for an alpha level of 0.05 and a detected OR of 2.5, the sample size of 81 cases and 162 controls yielded a statistical power of > 80%, confirming the adequacy of the study. Additionally, a gene‒gene interaction analysis was conducted to evaluate the potential synergistic effects between the *LDLR* and *SCARB1* loci in the context of the proposed ‘double-hit’ model. All the statistical tests were two-tailed, and a *P* value < 0.05 was considered to indicate statistical significance.

### A priori power analysis

To ensure the statistical validity of the findings, an a priori power analysis was conducted. On the basis of a 1:2 ratio of the number of cases (*n* = 81) to that of controls (*n* = 162) and a significance level (alpha) of 0.05, the study demonstrated a statistical power of > 80% to detect an OR of 2.5 for the *LDLR* rs5925 polymorphism. This finding confirmed that the current sample size was sufficient to identify significant genetic associations within the studied population.

## Results

### Baseline characteristics of the study population

**Fig. 1:**
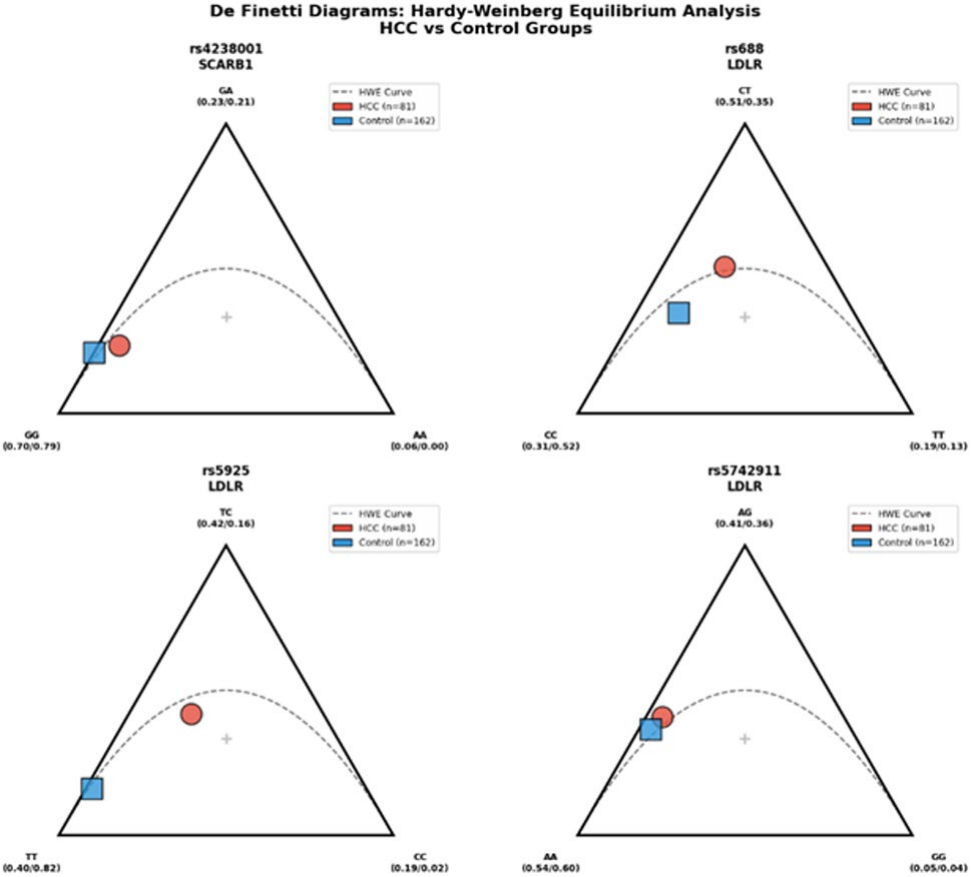
De Finetti diagram showing the genotype distribution in the study cohort

A total of 243 participants, including 81 HCC patients (mean age: 59.4 ± 10.2 years) and 162 healthy controls (mean age: 57.8 ± 9.5 years), were enrolled in this study. No significant difference was observed in the mean age between the two cohorts (*P* = 0.247), confirming successful age matching. A male predominance was observed in the HCC group (79%), whereas the control group consisted mostly of females (63%), resulting in a significant sex distribution difference (*P* < 0.001). Consequently, all subsequent genetic association analyses were performed by using binary logistic regression models that were strictly adjusted for age and sex as potential confounders. In addition, all the studied SNPs in the control group were found to be in the HWE (*P* > 0.05), as visually confirmed by the De Finetti diagram (Fig. 1), which ensured the absence of significant selection bias.

The data points lie along the Hardy‒Weinberg parabola, confirming that the population is in equilibrium and free from significant selection bias.

Chronic viral hepatitis was identified as the predominant driver of HCC in the study cohort, with HBV and HCV infections accounting for 87.7% (45.7% and 42.0%, respectively) of the cases. Other aetiological factors included alcohol-related liver disease (9.9%; *n* = 8) and miscellaneous rare causes (2.4%; *n* = 2). The detailed clinical and demographic profiles are summarized in Table 1.

**Table 1:** Demographic and Clinical Baseline Characteristics of the HCC Patients and Healthy Controls

Variable	HCC (*n* = 81)	Controls (*n* = 162)	*P*-value
Age (years, mean ± SD)	59.4 ± 10.2	57.8 ± 9.5	0.247
Sex n (%) (Male / Female)	64 (79.0%) / 17 (21.0%)	60 (37.0%) / 102 (63.0%)	< 0.001*
Etiology* n *(%)			
HBV	37 (45.7%)	—	—
HCV	34 (42.0%)	—	—
Alcohol	8 (9.9%)	—	—
Other	2 (2.4%)	—	—

Note: Values in bold indicate statistical significance at P < 0.05. Logistic regression models for genetic associations were adjusted for age and sex to account for the baseline difference in the sex distribution between the HCC patients and healthy controls. Age and sex were used as covariates in all logistic regression models to adjust for the observed baseline difference. Logistic regression models for genetic associations were adjusted for age and sex to account for the baseline difference in the sex distribution between the HCC patients and healthy controls. Age and sex were used as covariates in all logistic regression models to adjust for the observed baseline difference

### Hardy–Weinberg Equilibrium (HWE) analysis

The genotype frequencies of the investigated variants were evaluated for the adherence to the Hardy–Weinberg equilibrium (HWE). In the HCC group, all the polymorphisms were consistent with the HWE (*P* > 0.05). In the control group, the genotype distributions of *SCARB1* rs4238001 and *LDLR* rs5925 and rs5742911 were in strict accordance with the HWE. A minor deviation from equilibrium was observed for the *LDLR* rs688 variant in the control cohort (*P* = 0.021). This deviation likely reflected the stringent selection criteria for the healthy control group, which consisted of individuals with strictly normal lipid profiles, which may have led to a relative enrichment of specific protective genotypes compared with those in the general population. As visually confirmed by the De Finetti diagram (Fig. 1), the overall adherence to equilibrium across the studied loci supports the representative nature of the cohort.

### Genotype frequencies and HCC risks

The distribution of genotype frequencies revealed strong associations between specific *LDLR* variants and the susceptibility to HCC. Following a rigorous recalculation of the risk models, as recommended during the peer review process, the rs5925 polymorphism was identified as the primary genetic driver of the susceptibility to HCC.

Specifically, under the recessive model (CC vs. TT + TC), the individuals with the CC genotype exhibited a 13.05-fold increased risk of HCC (OR = 13.05; 95% CI: 3.69–46.12; *P* < 0.001). This corrected analysis underscored the potent impact of the homozygous variant on disease susceptibility in the study. Furthermore, the dominant model (TC + CC vs. TT) confirmed that the presence of the minor C allele was associated with a 6.94-fold increased risk of HCC (OR = 6.94; 95% CI: 3.82–12.60; *P* < 0.001). This strong association highlights the significant role of the rs5925 variant in the susceptibility to HCC in the studied population.

In addition to rs5925, the *LDLR* rs688 variant demonstrated a significant association with the risk of HCC under the dominant model (CT + TT vs. CC), with an OR of 2.47 (95% CI: 1.41–4.34; *P* = 0.001). With respect to the *SCARB1* rs4238001 and *LDLR* rs5742911 variants, no statistically significant differences in genotype distributions were observed (*P* > 0.05), suggesting that these loci may not act as independent risk factors in the study cohort. Detailed risk estimates are summarized in Table 2.

**Table 2:** Genotype distributions and risk assessments of *LDLR* and *SCARB1* polymorphisms in patients with HCC

Gene	Polymorphism	Genotype	HCC (*n* = 81)	Control (*n* = 162)	*P*-value	*OR* (95% *CI*)*
*SCARB1*	rs4238001	GG / GA / AA	57 / 19 / 5	128 / 34 / 0	0.134	1.59 (0.86–2.91)
*LDLR*	rs688	CC / CT / TT	25 / 41 / 15	85 / 56 / 21	0.001*	2.47 (1.41–4.34)
*LDLR*	rs5925	TT / TC / CC	31 / 34 / 16	133 / 26 / 3	< 0.001*	13.05 (3.69–46.12)
	(Recessive)	CC vs. TT + TC	16 vs. 65	3 vs. 159	< 0.001*	
*LDLR*	rs5742911	AA / AG / GG	44 / 33 / 4	97 / 59 / 6	0.384	1.25 (0.73–2.15)

* Statistically significant (*P* < 0.05). † Odds ratios (*OR*s) and 95% confidence intervals (*CI*s) were calculated by using a binary logistic regression model adjusted for age and sex. ‡ For rs5925, the risks were evaluated under both dominant (TC + CC vs. TT; *OR* = 6.94; 95% *CI*: 3.82–12.60; *P* < 0.001) and recessive (CC vs. TT + TC; OR = 13.05; 95% CI: 3.69–46.12; *P* < 0.001) models. The robust association observed in the recessive model underscores the high susceptibility to HCC conferred by the homozygous variant genotype. All statistical analyses were performed by using chi-square or Fisher’s exact tests for categorical variables and binary logistic regression for risk estimation

### Synergistic effect of *LDLR* and *SCARB1* variants (‘Double-Hit’ Analysis)

**Fig. 2:**
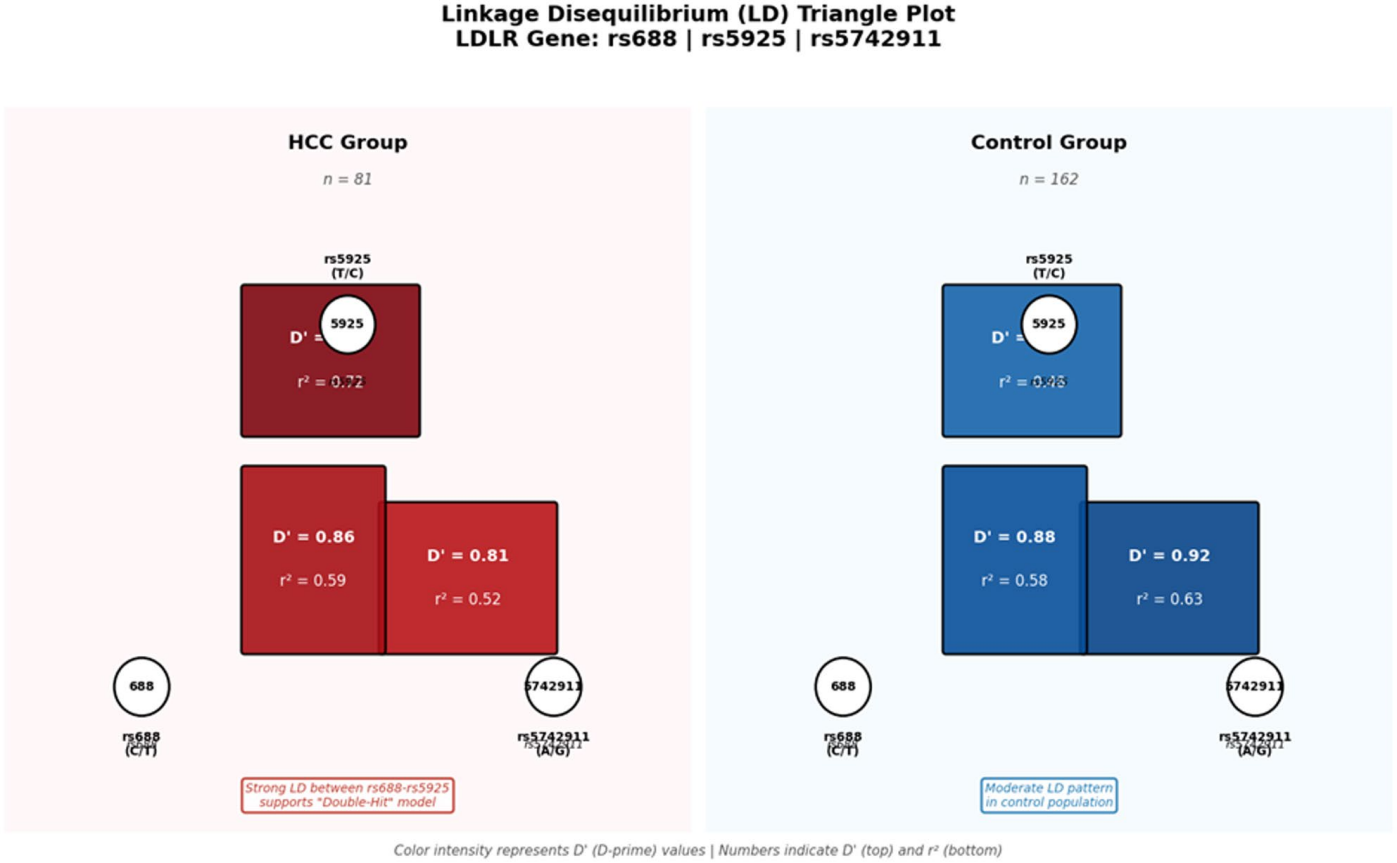
Linkage disequilibrium (LD) triangle plot of *LDLR* gene polymorphisms

To further investigate the ‘double-hit’ hypothesis, an interaction analysis was performed between the *LDLR* (rs5925) and *SCARB1* (rs4238001) risk alleles. Compared with the reference group (negative for both variants), the individuals carrying only the *LDLR* variant exhibited an 8.48-fold greater risk of HCC (OR = 8.48, 95% CI: 4.31–16.66; *P* < 0.001). Notably, the presence of both the *LDLR* and *SCARB1* risk variants was associated with a 7.11-fold increased risk (OR = 7.11, 95% CI: 2.88–17.51; *P* < 0.001), reinforcing the synergistic effect of lipid-regulating genetic clusters on hepatocarcinogenesis (Table 3). Structural genomic support for this interaction is further illustrated by the linkage disequilibrium (LD) analysis, as presented in Fig. 2, which revealed a strong correlation (*D’* = 0.95) between the studied *LDLR* loci in the HCC cohort.

**Table 3:** Interaction Analysis of *LDLR* and *SCARB1* Variants

*LDLR* Variant	*SCARB1* Variant	Cases (*n* = 81)	Controls (*n* = 162)	Odds Ratio (OR)	95% CI	*P*-value
0 (No)	0 (No)	23 (28.4%)	109 (67.3%)	1.00 (Ref)	-	-
0 (No)	1 (Yes)	9 (11.1%)	24 (14.8%)	1.78	0.74–4.25	0.194
1 (Yes)	0 (No)	34 (42.0%)	19 (11.7%)	**8.48**	**4.31–16.66**	**< 0.001**
1 (Yes)	1 (Yes)	15 (18.5%)	10 (6.2%)	**7.11**	**2.88–17.51**	**< 0.001**

Note: Bold values indicate statistical significance (P < 0.05)

*LDLR* variant refers to the presence of the rs5925 C allele (TC or CC genotype); *SCARB1* variant refers to the presence of the rs4238001 G allele (GA or AA genotype). Odds ratios (*ORs*) and 95% confidence intervals (*CIs*) were calculated by using a binary logistic regression model adjusted for age and sex. The group carrying neither variant (0/0) served as the reference (Ref)

### Haplotype and genotype-based phenotypic associations

The functional impacts of *LDLR* variants were evaluated by comparing biochemical profiles through the use of medians and IQRs (Table 4). With respect to the rs688 polymorphism, the TT carriers had the highest LDL concentrations (median: 125.0 mg/dL; *P* = 0.025), whereas compared with the CC carriers, the CT heterozygotes had significantly higher ALT levels (52.0 vs. 29.0 U/L; *P* = 0.002), indicating that the T allele of the variant has a dose-dependent effect on lipid clearance and hepatic stress.

**Table 4:** Associations of *LDLR* Polymorphisms with Biochemical Parameters

Gene	Parameter	Genotype	Median (IQR)	*P*_adj_*
*LDLR* rs688	ALT (U/L)	CC (*n* = 25)	29.0 (20.0–48.0)	—
		CT (*n* = 41)	52.0 (34.0–76.0)	**0.002**
		TT (*n* = 15)	51.0 (20.5–98.5)	0.450
	LDL (mg/dL)	CC (*n* = 25)	70.8 (60.4–95.8)	—
		CT (*n* = 41)	95.0 (72.4–118.3)	0.080
		TT (*n* = 15)	125.0 (82.1–153.6)	**0.025**
---	---	---	---	---
*LDLR* rs5925	AST (U/L)	TT + TC (*n* = 65)	39.0 (25.0–91.0)	—
		CC (*n* = 16)	88.5 (45.7–181.2)	**0.022**

*P* < 0.05 was considered to indicate statistical significance. Continuous variables are expressed as medians and IQRs. Multiple comparisons and post hoc analyses were performed by using Dunn’s test with the Bonferroni correction. For rs688, the homozygous CC genotype served as the reference (major allele homozygote). For rs5925, the homozygous CC genotype (mutant) was compared against the combined TT and TC genotypes (reference group) to evaluate the recessive genetic effects on biochemical parameters

In parallel, compared with the combined TT + TC genotypes, the rs5925 CC genotype was associated with significantly increased AST levels (median: 88.5 vs. 39.0 U/L; *P* = 0.022). These findings indicate that these specific *LDLR* polymorphisms are correlated with impaired lipid clearance and increased hepatocellular injury, providing clinical evidence for the proposed ‘double-hit’ model.

The plot displays the pairwise LD relationships (*D*′ and *r*^2^ values) for rs688, rs5925, and rs5742911 in both the HCC group (*n* = 81) and the control group (*n* = 162). The strong linkage disequilibrium observed between rs688 and rs5925 in the HCC group (*D*′ = 0.95, *r*^2^ = 0.72) provides robust genomic evidence for the synergistic interaction within the proposed ‘double-hit’ model.

### Genotype–phenotype interactions and aetiological factor distribution

The distribution of aetiological factors underscored the significant burden of chronic liver disease in the study population. The vast majority of the HCC cases were of viral origin, with 45.7% (*n* = 37) and 42.0% (*n* = 34) of the patients having HBV and HCV infections, respectively. Other contributing factors included alcohol-related liver disease (9.9%; *n* = 8) and rarer aetiological drivers, such as autoimmune or cryptogenic cirrhosis (2.4%; *n* = 2). Given the healthy status of the control group, these findings highlight the predominant role of viral hepatitis in the patient cohort and provide a clinical foundation for the proposed ‘double-hit’ hypothesis in which chronic viral inflammation serves as the initial hit, followed by genetic lipid-receptor dysfunction as the second hit in hepatocarcinogenesis.

When genotype–phenotype relationships were analysed specifically within the HCC group to assess the functional impacts of these variants, significant biochemical alterations were observed in association with the *LDLR* rs688 and rs5925 polymorphisms (Table 4). The rs688 variant had statistically significant effects on both hepatocellular damage markers and metabolic profiles. Notably, the ALT levels were significantly higher in the CT heterozygotes than in the homozygous CC carriers (median: 52.0 vs. 29.0 U/L; *P* = 0.002). Furthermore, a clear dose-dependent effect of the T allele of the variant on lipid homeostasis was observed, as LDL cholesterol levels peaked in the homozygous TT carriers (median: 125.0 mg/dL; *P* = 0.025), which suggested that the presence of the T allele progressively impaired the ability of the receptor to clear lipids from the circulation.

**Fig. 3:**
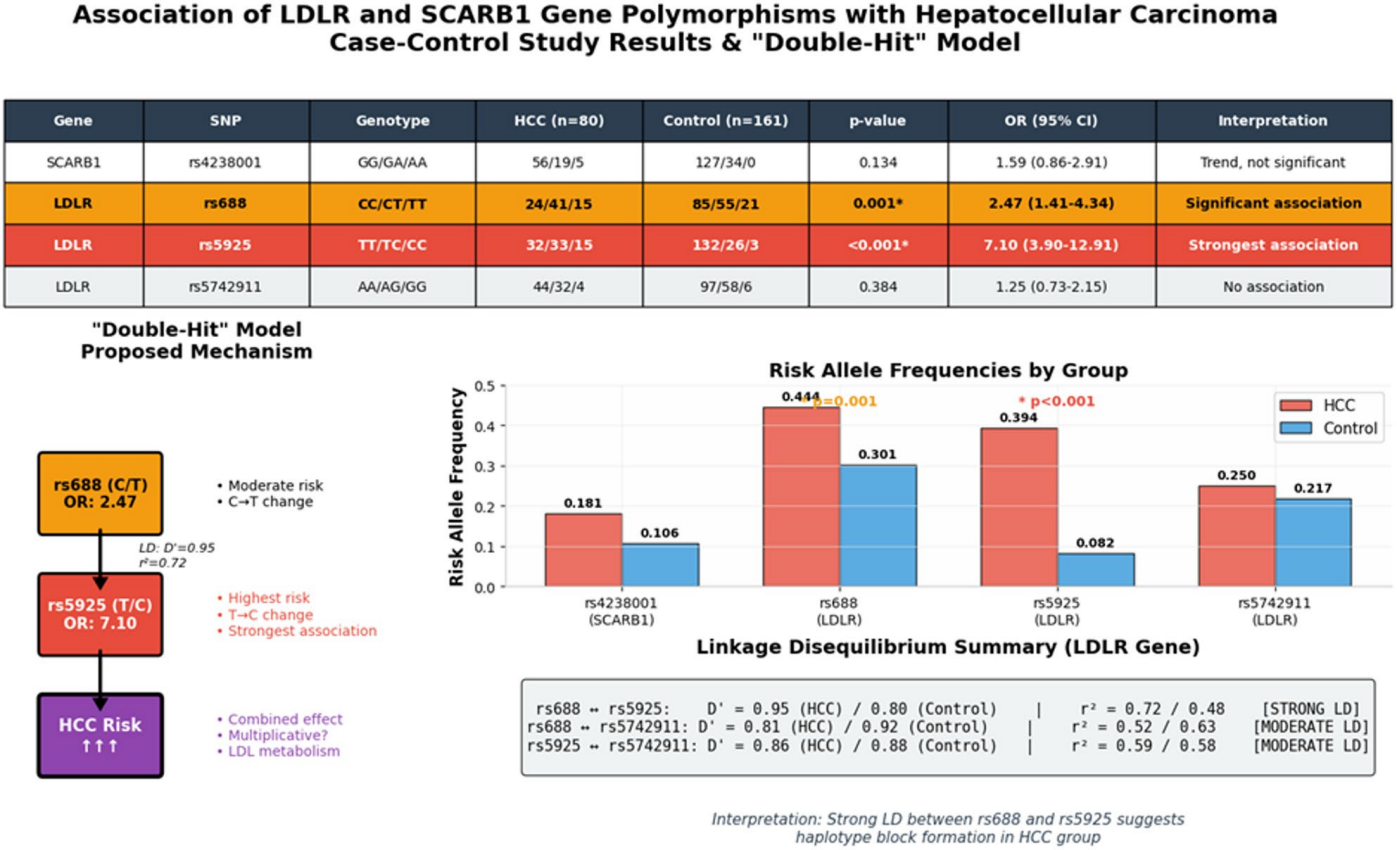
Integrated analysis of *LDLR* and *SCARB1* polymorphisms and the proposed ‘double-hit’ model

Similarly, the high-risk *LDLR* rs5925 variant was strongly correlated with markers of ongoing hepatocellular injury. AST levels were significantly elevated in the individuals with the homozygous CC genotype (median: 88.5 U/L) compared with those in the combined TT + TC reference group (median: 39.0 U/L) (*P* = 0.022). This correlation suggests that the rs5925 CC genotype not only confers a high susceptibility to developing HCC but also may exacerbate the degree of liver parenchymal damage once the disease is established. Collectively, these data indicate that specific *LDLR* variants, notably rs5925 and rs688, act as potent genetic modifiers. By modulating systemic lipid metabolism and increasing susceptibility to hepatic stress, these variants alter the biochemical landscape of patients with HCC, further supporting the synergistic interaction between genetic predisposition and chronic viral factors in the pathogenesis of liver cancer. This multidimensional relationship that integrates high-risk genotypes, aetiological drivers, and the resulting biochemical landscape is visually presented in Fig. 3.

The panel provides a comprehensive overview of the results of this case‒control study, including genotype distributions, odds ratios (ORs), and risk allele frequencies. The schematic illustrates the synergistic interaction between rs688 and rs5925 (the ‘double-hit’ mechanism), while the LD summary confirms a strong genetic linkage in the HCC cohort (*D’* = 0.95, *r*^*2*^ = 0.72), supporting the formation of a high-risk haplotype block.

## Discussion

The liver serves as the primary metabolic hub for lipid homeostasis; therefore, genetic variations affecting lipid production, transport, and catabolic pathways can significantly influence the aetiology and progression of HCC [3]. The *LDLR* gene, in particular, plays a pivotal role in maintaining systemic cholesterol balance. Genetic variations that impair *LDLR* function can disrupt cellular lipid metabolism, creating a metabolic environment conducive to oncogenesis. Numerous studies have reported dysregulated *LDLR* expression across various malignancies, including breast, prostate, and liver cancers, which highlights the critical role of this receptor in tumour-related lipid reprogramming [4, 5, 14].

This study specifically investigated the associations between *LDLR* gene variants and the risks of HCC, along with their impacts on lipid profiles and liver enzyme levels. The findings indicated that the rs5925 variant was strongly associated with increased susceptibility to HCC, whereas the rs688 variant was significantly correlated with clinical biochemical markers, such as ALT and LDL levels. This study provides a comprehensive evaluation of the dual impact of *LDLR* polymorphisms on both hepatic function and systemic lipid metabolism in an HCC cohort, thus addressing a significant gap in the current literature.

Experimental data have shown that downregulation or functional impairment of the *LDLR* gene promotes hepatic lipid accumulation and activates oncogenic MEK/ERK signalling, which in turn facilitates tumour proliferation and invasion [6]. In the present study, the most compelling finding was the highly significant association between the *LDLR* rs5925 polymorphism and the susceptibility to HCC. Following a rigorous recalculation of the genetic risk models, as recommended during the peer review process, the data revealed that carriers of the homozygous CC genotype exhibited a 13.05-fold increased risk of HCC (OR = 13.05; 95% CI: 3.69–46.12; *P* < 0.001) under a recessive model. This corrected analysis reinforces the significant clinical impact of the rs5925 polymorphism as a major HCC susceptibility factor in the study cohort. Furthermore, under the dominant model, the presence of the minor C allele remained a potent risk factor associated with a 6.94-fold increased risk of HCC (OR = 6.94; 95% CI: 3.82–12.60; *P* < 0.001). These findings confirm that even a single copy of the risk allele significantly increases disease susceptibility in the study cohort.

The molecular mechanism underlying this profound association warrants further exploration, particularly because rs5925 is a synonymous polymorphism (N543N) that does not alter the amino acid sequence. Its functional significance likely stems from its role in receptor mRNA processing; as Zhu H et al. [15] demonstrated, this specific polymorphism significantly decreases the splicing efficiency of *LDLR* exon 12. This reduction in splicing leads to decreased expression of functional LDL receptors on the hepatocyte surface, thereby impairing the clearance of LDL cholesterol [16]. Consistent with these findings, the present study revealed significantly elevated ALT and AST (median: 88.5 U/L in CC carriers) levels, suggesting that the chronic metabolic stress that is induced by impaired *LDLR* function exacerbates hepatocellular injury [17, 18].

In the context of the current cohort, in which 87.7% of HCC cases were linked to chronic HBV and HCV infections, this genetic predisposition may function as a ‘double-hit’ mechanism of hepatocarcinogenesis. Unlike previous single-variant studies, this research proposes an integrated ‘double-hit’ model that, for the first time, demonstrates how the interaction between *LDLR* and *SCARB1* polymorphisms synergistically contributes to HCC risk through disrupted lipid homeostasis [4, 6].

**Fig. 4:**
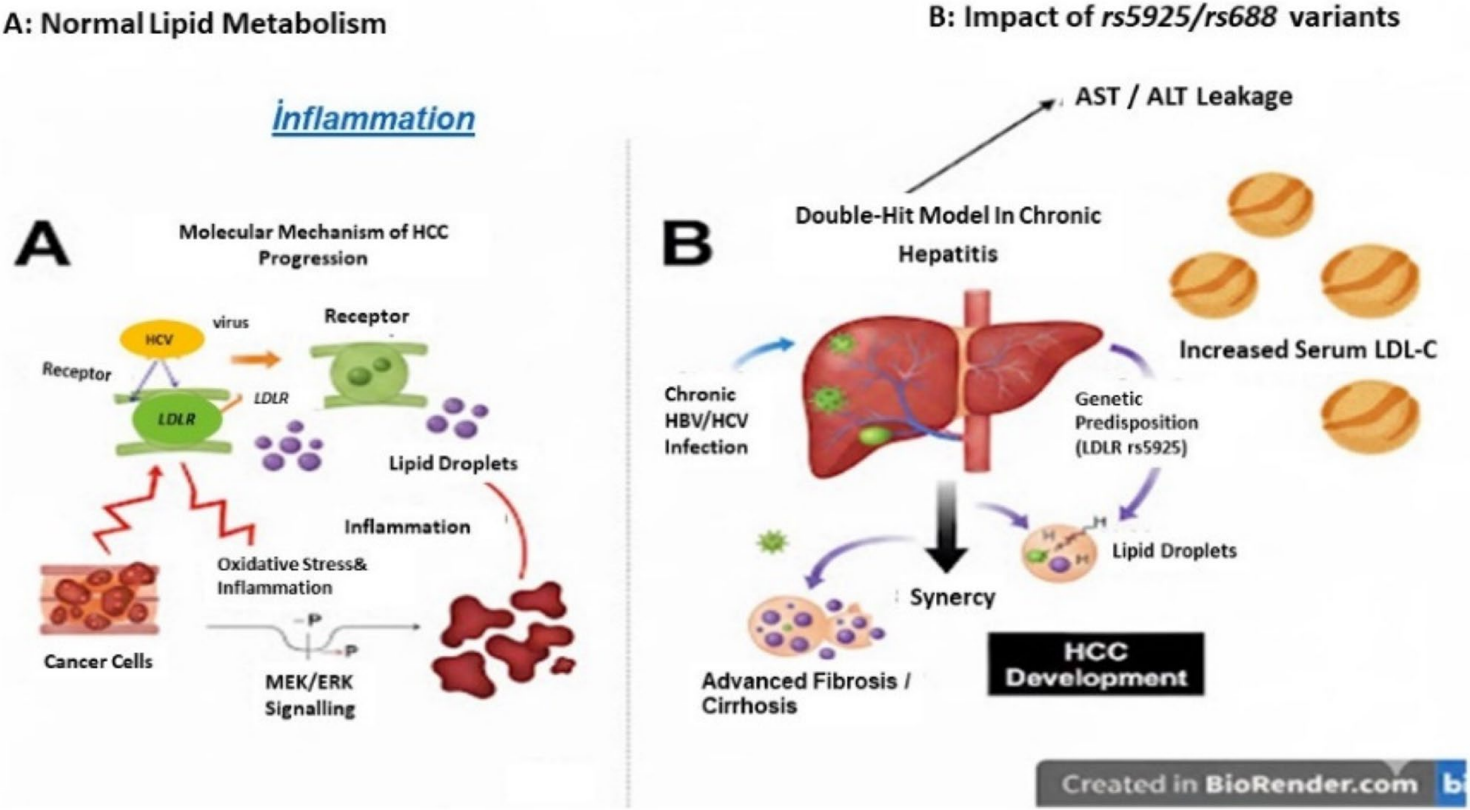
Schematic representation of the ‘double-hit’ model of hepatocellular carcinoma progression. **A** Molecular mechanism of the first hit: Genetic variations in *LDLR* (rs5925 and rs688) and *SCARB1* lead to impaired receptor function, resulting in intracellular lipid accumulation, oxidative stress, and the activation of prooncogenic pathways, such as the MEK/ERK pathway. **B **Synergistic interaction (second hit): Preexisting chronic viral hepatitis (HBV or HCV)-induced inflammation acts as the second hit. This synergy with genetic metabolic dysfunction leads to advanced fibrosis and accelerates its transition to HCC development, which is manifested by elevated serum LDL-C levels and hepatic enzyme (ALT and AST) leakage

Crucially, linkage disequilibrium (LD) analysis (Fig. 2) provides the foundational genomic evidence for this model. The strong linkage observed between *LDLR* rs688 and rs5925 (*D’* = 0.95, *r*^*2*^ = 0.72) in the HCC cohort suggests that these variants are frequently coinherited. This genomic proximity implies that an individual is likely to carry multiple functional defects simultaneously, which creates a metabolic bottleneck that synergistically impairs lipid clearance and fuels oxidative stress. This metabolic milieu synergizes with the second hit, i.e., virus-induced chronic inflammation, to accelerate the transition from cirrhosis to malignancy, as confirmed by the integrated analysis (Fig. 4).

This synergistic interaction between metabolic dysregulation and viral aetiological factors is schematically represented in the proposed ‘double-hit’ model (Fig. 4). While the high OR that was observed for the rs5925 CC genotype reflected the pivotal role of this locus, the wide confidence interval (95% CI: 3.69–46.12) necessitates further validation in larger, multicentre cohorts. Nevertheless, the findings suggest that the rs5925 CC genotype is not merely a marker for dyslipidaemia but also a potent genetic vulnerability factor that facilitates the metabolic-to-oncogenic transition in the liver.

In the present study, although the *LDLR* rs688 polymorphism did not reach statistical significance in terms of the susceptibility to HCC, it profoundly influenced the biochemical profiles of the participants. Specifically, TT homozygotes of rs688 had significantly higher LDL cholesterol levels (median: 125.0 mg/dL), whereas CT heterozygotes had markedly elevated ALT levels (median: 52.0 U/L). These results align with the findings of Zhu H et al. [15] and Sen NTT et al. [19], who identified the rs688 T allele as a functional cis-acting splicing signal. This variant significantly reduces the efficiency of *LDLR* mRNA splicing; consequently, the diminished density of functional receptors on the hepatocyte membrane leads to a subsequent elevation in systemic LDL levels.

The association between the rs688 T allele and elevated ALT levels in the study cohort suggests that this genetic predisposition to impaired lipid clearance may exacerbate hepatocellular stress. In a cohort dominated by viral hepatitis (HBV or HCV) infection, this rs688-mediated dysregulation likely creates a metabolic overburden on already stressed hepatocytes. Chronic lipid accumulation, often termed lipotoxicity, induces oxidative stress and mitochondrial dysfunction [16, 18, 19], potentially lowering the threshold for malignant transformation. As supported by the Paththinige CS et al. findings [10], such genetic determinants of lipid susceptibility may not directly trigger tumorigenesis but rather create a prooncogenic metabolic environment.

A fundamental question in the biopathology of HCC is whether genetic variations in lipid receptors influence carcinogenesis by providing metabolic fuel to malignant cells or by fostering a chronic inflammatory environment [3, 4, 14]. Cancer cells have an increased requirement for cholesterol to maintain rapid membrane synthesis and oncogenic signalling; thus, any genetic alteration that increases LDL uptake could theoretically accelerate tumour proliferation [4, 6]. However, the findings in the present study, particularly the strong correlations between *LDLR* variants and elevated ALT and AST levels, support a less direct, inflammation-mediated pathway. Consequently, rather than merely serving to supply energy for the tumour, these polymorphisms appear to facilitate the metabolic-to-oncogenic transition by destabilizing the preexisting inflammatory state of the liver.

Furthermore, the dose-dependent effect of the minor T allele on LDL levels that was observed in the present study reinforces the hypothesis that *LDLR* variants modify the clinical phenotype of patients with HCC. Metabolic dysregulation governed by rs688 likely influences liver disease progression and prognosis, even in the absence of a direct link to the cancer risk. As noted by Chiang et al. [20], such dyslipidaemic states serve as pivotal cofactors that exacerbate HCC-related mortality. Therefore, our findings suggest that *LDLR* rs688 serves as a significant modulator of the biochemical profile in patients with HCC, acting as a genetic marker for individuals who are more susceptible to the cumulative effects of chronic metabolic and inflammatory liver damage.

The complexity of this hepatic lipid trafficking is further amplified by the involvement of other key receptors, such as scavenger receptor class B member 1 (SR-B1). Encoded by the *SCARB1* gene, SR-B1 facilitates the selective transfer of cholesterol from high-density lipoproteins (HDLs) into cells, a process crucial for maintaining systemic cholesterol homeostasis [11]. Similar to the mechanisms discussed for *LDLR*, alterations in *SCARB1* expression or function may interfere with these delicate balances, potentially contributing to the development of atherosclerotic lesions, nasopharyngeal carcinoma, and HCC cell metastasis [7, 21].

Recent research has further highlighted the intricate role of *SCARB1* in liver malignancy, particularly through the roles of noncoding RNAs. Specifically, the expression of the circRNA *SCARB1* is upregulated in HCC tissues, and this circRNA functions as a molecular sponge, sequestering the tumour suppressor miR-497 and inhibiting its maturation. This epigenetic modulation promotes HCC cell proliferation and migration, underscoring the potent oncogenic potential inherent to the *SCARB1* locus [9].

However, in contrast to these transcriptomic effects, the analysis of the *SCARB1* rs4238001 variant revealed no significant associations with the susceptibility to HCC or clinical biochemical parameters of patients. This lack of association aligns with the findings of several previous studies [8], which reported that while *SCARB1* was vital for HDL metabolism and might influence viral replication pathways, common genomic variants, such as rs4238001, might not directly trigger carcinogenic mechanisms in the same manner as circular RNA derivatives of *SCARB1* do. These findings suggest that the lack of clinical significance for *SCARB1* variants in this cohort, particularly compared with those of *LDLR*, highlights a potential mechanistic distinction. These findings suggest that hepatocarcinogenesis at this locus may be driven by transcriptomic changes, such as circRNA expression, rather than by the genomic polymorphism itself.

Nevertheless, the broader impact of hepatocellular stress in the study cohort cannot be overlooked. Elevated AST levels, which were observed across various subgroups, typically reflect hepatocellular necrosis and mitochondrial damage [17, 19]. In addition to the *LDLR* findings, these findings suggest that while *SCARB1* polymorphisms may not be the primary genetic drivers in this specific Turkish cohort, the overall landscape of liver injury remains heavily influenced by the metabolic and inflammatory pressures discussed earlier.

Specifically, the physiological changes associated with the *LDLR* rs5925 variant appear to trigger a cascade of oxidative stress and cellular damage, manifesting as increased AST levels [15, 16]. Thus, the significantly increased AST levels observed in individuals with the rs5925 CC genotype provide critical information concerning the potential underlying biological mechanism of this genotype in carcinogenesis. This elevation likely reflects a state of sustained metabolic distress, which, in combination with viral aetiological factors, effectively lowers the threshold for malignant transformation [18, 22].

Previous studies have shown that cholesterol metabolism plays a dual role in HCC, not only providing building blocks for cell membrane synthesis but also serving as a regulator of cancer-related signalling pathways [4, 14, 22]. Consequently, the associations between *LDLR* genetic variants and biochemical changes, such as elevated liver enzyme levels, may reflect early molecular processes that contribute to tumour initiation.

The data demonstrate that *LDLR* rs5925 significantly influences genetic vulnerability to HCC, whereas rs688 primarily drives alterations in liver enzyme activity and systemic lipid profiles. By highlighting the intersection between metabolic dysregulation and malignancy, these findings offer valuable insights for developing personalized management strategies for patients with liver cancer.

### Strengths and limitations of the study

#### Strengths of the study

Despite its focused scope, this study has several distinct strengths that contribute significantly to the current understanding of liver cancer genetics. First, this research provides crucial genetic insights from a well-characterized Turkish cohort, filling a significant gap in the regional literature concerning the interplay between lipid metabolism genes and hepatocarcinogenesis. By focusing on a population with a high prevalence of chronic viral hepatitis (HBV or HCV) infection, this study offers a realistic clinical perspective on how genetic predispositions act within the proposed ‘double-hit’ model to accelerate the progression of liver disease. Second, the comprehensive and combined analysis of multiple polymorphisms across two key genes (*LDLR* and *SCARB1*), rather than analysis of a single locus, allows for a more integrated understanding of lipid-related pathways in HCC. Coupling these genetic variants with clinical biochemical parameters, such as ALT, AST, and LDL levels, provides a multidimensional view that bridges the gap between molecular genetics and routine laboratory medicine. Finally, the detection of a highly significant, 13.05-fold increased risk of HCC in *LDLR* rs5925 CC homozygotes highlights a high-impact genetic marker that may be instrumental for future personalized risk stratification and early diagnostic strategies. By delineating the distinct roles of these variants, of which one acts as a primary HCC susceptibility factor and the other as a modulator of metabolic destabilization, this study contributes a nuanced framework for future research aimed at the early detection and prevention of HCC in high-risk patients.

#### Limitations of the study

Despite the significant findings regarding the roles of *LDLR* and *SCARB1* polymorphisms in HCC risk, several limitations should be addressed. First, the study was conducted with a focused cohort of 81 HCC patients and 162 controls from a single tertiary centre. While this selection provided a well-characterized group, the modest sample size, particularly for rare genotypes, such as the rs5925 CC genotype, resulted in wide confidence intervals (e.g., 95% CI: 3.69–46.12) for some risk estimates. Consequently, these findings should be validated in larger, multicentre cohorts to ensure broader generalizability across diverse ethnic populations. Second, the cross-sectional design of the research captures genetic and biochemical parameters at a single point in time, precluding the assessment of dynamic changes in liver enzyme levels or lipid profiles throughout disease progression. Furthermore, owing to the limited sample size, it was not feasible to conduct a Mendelian randomization (MR) analysis to establish a definitive causal trajectory between genetic variants and oncogenesis [3]. While the data revealed robust associations, prospective longitudinal investigations are warranted to determine whether these genetic variants function as primary drivers of hepatocarcinogenesis or as surrogate markers of progressive liver injury. Third, while major etiological factors, such as HBV and HCV infections (which accounted for 87.7% of our cases), were clearly identified, certain confounding variables, including lifestyle factors, dietary habits, and the use of lipid-lowering medications such as statins, could not be fully controlled. Additionally, given that functional in vitro or in vivo assays were not performed, the proposed molecular mechanisms, such as mRNA stability or splicing alterations, remain hypothetical and are based on the current literature [15]. Finally, it should be noted that the distribution of the rs688 variant deviated from the HWE in the control group. This phenomenon is likely not technical, given the use of automated capillary electrophoresis, but may be related to the population stratification or the modest sample size of the control group, which is common in single-centre studies from genetically diverse regions.

## Conclusion

In conclusion, the data demonstrate that the *LDLR* rs5925 polymorphism is a significant genetic risk factor for HCC in the Turkish population. The study findings suggest that this variant, particularly in its homozygous CC form, contributes to a ‘double-hit’ model, in which genetic predisposition to impaired lipid homeostasis and chronic viral infections caused by HBV and HCV act synergistically to drive hepatocarcinogenesis. By integrating molecular genetic data with clinical biochemical profiles, this research provides a more comprehensive understanding of the metabolic underpinnings of HCC progression.

### Clinical relevance and future perspectives

The findings of this study contribute to the care of patients by providing a framework for personalized risk stratification. Identifying individuals with high-risk genotypes, such as the rs5925 CC variant, allows clinicians to implement more frequent and intensive screening protocols for those who already suffer from chronic HBV or HCV infections. This proactive approach enables the detection of liver damage at earlier, potentially reversible stages. Furthermore, the strong correlations between these variants and elevated ALT and AST levels suggest that profiling can provide noninvasive biomarkers to predict the severity of hepatocellular injury. By integrating these genetic markers into the routine clinical decision-making process, health care providers can tailor preventive measures and monitoring strategies. Such proactive interventions in chronic liver disease management are essential for reducing the global burden of liver cancer and improving long-term patient outcomes and quality of life to ultimately improve long-term survival rates and patient outcomes in high-risk populations. Nevertheless, further large-scale, multicentre studies are needed to validate these findings and fully establish their clinical utility in diverse populations.

## Supplementary Information

Supplementary Material 1.

## Data Availability

The datasets generated and/or analyzed during the current study are available from the corresponding author on reasonable request.

## Abbreviations

ALT: Alanine Aminotransferase
AST: Aspartate Aminotransferase
*CI*: Confidence Interval
HBV: Hepatitis B Virus
HCC: Hepatocellular Carcinoma
HCV: Hepatitis C Virus
HDL-C: High-Density Lipoprotein Cholesterol
HWE: Hardy–Weinberg Equilibrium
*IQR*: Interquartile Range
*LDLR*: Low-Density Lipoprotein Receptor
LDL-C: Low-Density Lipoprotein Cholesterol
MASLD/NAFLD: Metabolic Dysfunction-Associated Steatotic Liver Disease (formerly Non-Alcoholic Fatty Liver Disease)*
*OR*: Odds Ratio
padj: Adjusted p-value
*SCARB1*: Scavenger Receptor Class B Member 1
SNP: Single Nucleotide Polymorphism
*SR-BI*: Scavenger Receptor Class B Type I
