# Supplementary material for: Association of LDLR and SCARB1 gene polymorphisms with hepatocellular carcinoma: a case-control study proposing a ‘double-hit’ model
Source: Lipids Health Dis. 2026 Apr 11;25:136. doi: 10.1186/s12944-026-02946-x (PMC13188632; doi:10.1186/s12944-026-02946-x)
Supplement: Supplementary file 1 — Supplementary Material 1. [file 12944_2026_2946_MOESM1_ESM.pdf]

SPRINGER NATURE
Author Services

Editing Certificate

This document certifies that the manuscript

"Association of LDLR and SCARB1 Gene Polymorphisms with Hepatocellular Carcinoma: A Case-Control Study Proposing a ‘Double-Hit’ Model"

prepared by the authors

Zuhal ALTINTAS,Muammer Ozgur CEVIK, Ebru DERICI EKER, Engin ALTINTAS

was edited for proper English language, grammar, punctuation, spelling, and overall style by one or more of the highly qualified English speaking editors at SNAS.

This certificate was issued on **March 26, 2026** and may be verified on the SNAS website using the verification code **ED97-DFDC-39A5-1356-7539**.

Neither the research content nor the authors' intentions were altered in any way during the editing process. Documents receiving this certification should be English-ready for publication; however, the author has the ability to accept or reject our suggestions and changes. To verify the final SNAS edited version, please visit our verification page at secure.authorservices.springernature.com/certificate/verify. If you have any questions or concerns about this edited document, please contact SNAS at support@as.springernature.com.

SNAS provides a range of editing, translation, and manuscript services for researchers and publishers around the world. For more information about our company, services, and partner discounts, please visit authorservices.springernature.com.

itenticate Association of LDLR and SCARB1 Gene Polymorphisms with Hepatocellular Carcinoma A Case-Control...

Mersin Üniversitesi

Document Details

Submission ID

trn:oid:::3117:570216404

Submission Date

Mar 23, 2026, 10:12 AM GMT+3

Download Date

Mar 23, 2026, 10:14 AM GMT+3

File Name

itenticate Association of LDLR and SCARB1 Gene Polymorphisms with Hepatocellular Carcinoma....doc

File Size

536.5 KB

27 Pages

6,225 Words

37,107 Characters

11% Overall Similarity

The combined total of all matches, including overlapping sources, for each database.

Match Groups

- **70** Not Cited or Quoted 11%
  Matches with neither in-text citation nor quotation marks
- **0** Missing Quotations 0%
  Matches that are still very similar to source material
- **1** Missing Citation 0%
  Matches that have quotation marks, but no in-text citation
- **0** Cited and Quoted 0%
  Matches with in-text citation present, but no quotation marks

Top Sources

10% Internet sources
4% Publications
0% Submitted works (Student Papers)

Integrity Flags

0 Integrity Flags for Review

No suspicious text manipulations found.

Our system's algorithms look deeply at a document for any inconsistencies that would set it apart from a normal submission. If we notice something strange, we flag it for you to review.

A Flag is not necessarily an indicator of a problem. However, we'd recommend you focus your attention there for further review.

Match Groups

70 Not Cited or Quoted 11%
Matches with neither in-text citation nor quotation marks

0 Missing Quotations 0%
Matches that are still very similar to source material

1 Missing Citation 0%
Matches that have quotation marks, but no in-text citation

0 Cited and Quoted 0%
Matches with in-text citation present, but no quotation marks

Top Sources

10% Internet sources
4% Publications
0% Submitted works (Student Papers)

Top Sources

The sources with the highest number of matches within the submission. Overlapping sources will not be displayed.

#	Type	Source	Match
1	Internet	www.mdpi.com	2%
2	Internet	www.frontiersin.org	1%
3	Internet	www.researchsquare.com	<1%
4	Internet	worldwidescience.org	<1%
5	Internet	www.researchgate.net	<1%
6	Internet	ndl.ethernet.edu.et	<1%
7	Internet	public-pages-files-2025.frontiersin.org	<1%
8	Internet	www.science.gov	<1%
9	Publication	Ajeet Singh Bhadoria, Samiran Nundy. "Preventive Hepatology", CRC Press, 2025	<1%
10	Internet	www.jurolsurgery.org	<1%

11 Publication

Man Kit Christopher Chu, Man Tong. "Emerging potential of CAR-NK cell therapy f... <1%

12 Internet

www.newjournalurology.com <1%

13 Internet

d.docksci.com <1%

14 Internet

ecerm.org <1%

15 Internet

iscoms.com <1%

16 Internet

medsci.org <1%

17 Internet

rcastoragev2.blob.core.windows.net <1%

18 Publication

Hamid D. Ismail. "Bioinformatics of Autoimmune Diseases", CRC Press, 2026 <1%

19 Internet

pmc.ncbi.nlm.nih.gov <1%

20 Internet

assets-eu.researchsquare.com <1%

21 Internet

www.jneonatalsurg.com <1%

22 Internet

ousar.lib.okayama-u.ac.jp <1%

23 Internet

jped.elsevier.es <1%

24 Internet

oxfordjournals.org <1%

25 Internet

www.sciencepub.net <1%

26 Publication

Van Khanh Nguyen, Kosuke Matsui, Hisashi Kosaka, Hideyuki Matsushima et al. "... <1%

27 Internet

eandv.biomedcentral.com <1%

28 Internet

www.tjn.org.tr <1%

29 Internet

pdfs.semanticscholar.org <1%

30 Publication

Cummings, Meredith Hughes Collier. "Temporal Patterns of Symptoms During Tr... <1%

31 Publication

Thomas P.A. Debray, Tri-Long Nguyen, Robert W. Platt. "Comparative Effectivenes... <1%

32 Internet

arthritis-research.biomedcentral.com <1%

33 Internet

assets.cureus.com <1%

34 Internet

effiloop.com <1%

35 Internet

jzusb.zjujournals.com <1%

36 Internet

www.dbc.wroc.pl <1%

37 Internet

www.koreascience.kr <1%

Abstract

Background: The Low-Density Lipoprotein Receptor (*LDLR*) and Scavenger Receptor Class B Member 1 (*SCARB1*) are essential regulators of systemic lipid homeostasis. Although dysregulated lipid metabolism is a hallmark of malignancy, the genetic association between these receptors and hepatocellular carcinoma (HCC) risk remains poorly understood. This study aimed to evaluate the impact of *LDLR* and *SCARB1* polymorphisms on HCC susceptibility and clinical biochemical profiles in a population with high viral hepatitis prevalence.

Methods: In this case-control study, 81 HCC patients and 162 healthy controls were genotyped for *SCARB1* rs4238001 and *LDLR* rs688 and rs5925 variants using an automated capillary electrophoresis system. Genetic data were correlated with clinical markers of liver injury, including alanine aminotransferase (ALT) and aspartate aminotransferase (AST) levels, as well as low-density lipoprotein cholesterol (LDL-C). Statistical analyses utilized logistic regression for risk estimation and non-parametric tests (Median and Interquartile Range) for biochemical correlations.

Results: The LDLR rs5925 polymorphism emerged as a major risk factor for HCC. Under the recessive model, the homozygous CC genotype exhibited a 13.05-fold increased risk of HCC compared to the reference genotypes (OR=13.05, 95% CI: 3.69–46.12, $P < 0.001$). Under the dominant model, carriers of the minor C allele had a 6.94-fold higher risk (OR=6.94, 95% CI: 3.82–12.60, $P < 0.001$). Additionally, the *LDLR* rs688 variant was linked to metabolic alterations; TT homozygotes showed significantly higher LDL-C levels (p=0.025), while CT heterozygotes exhibited elevated ALT ($P = 0.002$). Notably, the rs5925 CC genotype strongly correlated with elevated AST ($P = 0.022$), suggesting a synergistic link between genetic predisposition and hepatocellular injury. Building on these findings, the interaction analysis supports an integrated 'Double-Hit' model, demonstrating for the first time how the synergy between *LDLR* and *SCARB1* polymorphisms modulates HCC risk through disrupted lipid homeostasis.

Conclusion: The findings demonstrate demonstrate that the *LDLR* rs5925 variant is a potent genetic marker for HCC susceptibility. The association of *LDLR* variants with impaired lipid homeostasis and hepatic stress supports a 'Double-Hit' model, where genetic vulnerability and chronic viral factors synergistically promote hepatocarcinogenesis. These results highlight the potential utility of genetic screening for personalized risk stratification in patients with chronic liver disease.

Keywords: Genetic polymorphism; Hepatocellular carcinoma (HCC); Lipid metabolism; Liver enzymes; Low-density lipoprotein receptor (LDLR); rs5925; *SCARB1*

1. Introduction

Hepatocellular carcinoma (HCC) is the predominant primary liver malignancy and ranks as the second leading cause of cancer-related mortality worldwide [1, 2]. The global incidence of HCC is steadily rising, particularly in regions with a high prevalence of chronic viral

hepatitis, such as hepatitis B virus (HBV) and hepatitis C virus (HCV) infections. While established risk factors, including viral hepatitis, chronic alcohol consumption, metabolic syndrome, obesity, and non-alcoholic fatty liver disease (NAFLD), drive the majority of cases, emerging evidence underscores the critical role of lipid metabolism dysregulation in hepatocarcinogenesis [1, 3, 4].

As the central hub for cholesterol synthesis and metabolism, the liver maintains systemic lipid homeostasis. Cholesterol is not only a vital structural component of cell membranes but also a precursor for signaling molecules. In HCC, the metabolic landscape is frequently reprogrammed; tumor cells often exhibit decreased cholesterol excretion alongside accelerated uptake and endogenous production. This metabolic shift fosters a pro-inflammatory environment, induces lipotoxicity, and promotes hepatic fibrosis, all of which are precursors to malignant transformation [4, 5].

LDLR is a key modulator of this process, mediating the endocytosis of cholesterol-rich LDL particles from the systemic circulation. Polymorphisms within the *LDLR* gene can significantly impair receptor efficiency, leading to abnormal lipid accumulation in the liver and elevated systemic levels [4]. Experimental models have established that *LDLR* dysfunction serves as a critical catalyst for hepatocarcinogenesis by triggering intracellular cholesterol sequestration and chronic oxidative stress. This metabolic disruption subsequently activates oncogenic cascades, most notably the MEK/ERK signaling pathway, thereby establishing a mechanistic link between systemic lipid disorders and the molecular initiation of tumorigenesis [6]. Similarly, the Scavenger Receptor Class B Member 1 (*SCARB1*) gene facilitates selective cholesterol uptake and efflux. The dysregulation of has been implicated in both HCC tumor progression and the viral replication cycles of HBV and HCV [7 - 9].

Despite the established roles of lipid metabolism in hepatic health, the collective impact of *LDLR* and *SCARB1* genetic variations on HCC risk, particularly in populations with high viral hepatitis prevalence, remains poorly characterized. Most existing studies have focused on single-variant associations, often overlooking the complex interplay between metabolic genetic predispositions and viral etiological factors. Therefore, this study was designed to evaluate the synergistic influence of *LDLR* and *SCARB1* polymorphisms on HCC susceptibility and clinical biochemical profiles. The central hypothesis is that these genetic variants do not act in isolation but rather function through a 'Double-Hit' mechanism, where impaired lipid homeostasis and chronic viral inflammation converge to accelerate hepatocarcinogenesi. By integrating genomic data with real-world clinical parameters, the current research aims to provide a novel multidimensional framework for personalized risk stratification in patients with chronic liver disease.

2. Materials and Methods

2.1. Study Population and Ethical Statement

This case-control study was designed to investigate the association between *LDLR* and *SCARB1* gene polymorphisms and the risk of developing HCC. The study population comprised 81 patients diagnosed with HCC and 162 age- and gender-matched healthy controls. All participants were recruited from the Department of Gastroenterology at Mersin University between 2023 and 2024.

The HCC group comprised individuals over 18 years of age with a diagnosis established in accordance with the European Association for the Study of the Liver (EASL) guidelines [10]. Utilizing multiphasic computed tomography (CT), magnetic resonance imaging (MRI), and clinical or histological confirmation where indicated, patients were monitored between 2023 and 2024. The control group consisted of healthy individuals with no prior history of

malignancy, metabolic disorders, or chronic liver disease, serving as a baseline for genetic and biochemical comparisons.

The study protocol was conducted in strict accordance with the ethical principles of the Declaration of Helsinki [11]. The research was initiated following formal approval from the local Ethics Committee on November 15, 2023 (Decision No: 2023/781). This primary approval covered the initial participant recruitment and sample collection period between 2023 and 2024. To ensure comprehensive coverage of the advanced genetic evaluations and the specific 'Double-Hit' interaction model analyses, an administrative update and extension to the protocol were finalized in 2025 (Ref No: 2025/529). This sequential approval process ensured that all stages of the investigation, from recruitment to specialized genetic data processing, were performed under rigorous institutional oversight.

2.2. Biochemical and Clinical Data Collection

Demographic information, liver function tests (ALT, AST, ALP, GGT), and complete blood counts were extracted from the hospital’s electronic medical record system for all participants. All clinical measurements were performed using standardized laboratory techniques before the initiation of any therapeutic interventions to ensure baseline consistency. In accordance with the distribution of the biochemical data, values were reported as medians and interquartile ranges (IQR) to maintain statistical accuracy during comparisons.

2.3. Genotyping and DNA Isolation

Whole blood samples were collected into vacuum tubes containing ethylenediaminetetraacetic acid (EDTA) as an anticoagulant and stored at -20°C until analysis. Genomic DNA extraction was performed using the PureLink Genomic DNA Isolation Kit (Invitrogen) based on the spin column method, following the manufacturer instructions. The quantity and purity of the extracted DNA were validated via spectrophotometric analysis.

The analysis of the genetic variants, *SCARB1* rs4238001, *LDLR* rs688, rs5925, and rs5742911 was performed using the Polymerase Chain Reaction-Restriction Fragment Length Polymorphism (PCR-RFLP) technique. Following the restriction enzyme digestion, the resulting DNA fragments were analyzed using the QIAxcel Advanced System (Qiagen), an automated capillary electrophoresis platform. This system provided high-resolution fragment separation and precise genotype callings through digital data analysis, ensuring significantly higher accuracy and reproducibility compared to traditional manual agarose gel electrophoresis.

2.4. Statistical Analysis

Statistical analyses were conducted using IBM SPSS Statistics version 29.0 and Python 3.11. The normality of the data distribution was evaluated using the Kolmogorov–Smirnov test. Descriptive statistics for continuous variables were expressed as mean ± standard deviation or median (interquartile range [IQR]), while categorical variables were expressed as frequencies and percentages.

For continuous data, the Kruskal–Wallis and Mann–Whitney U tests were employed for non-parametric comparisons. Genotype-phenotype relationships and the distribution of allele frequencies were evaluated using the Pearson chi-square (χ^2) test. Odds ratios (OR) and 95% confidence intervals (CI) were calculated using a binary logistic regression model, adjusted for age and gender as potential confounders. The adherence of genotype frequencies to the Hardy-Weinberg Equilibrium (HWE) was tested in the control group.

Following reviewer suggestions, an a priori power analysis was performed; for an alpha level of 0.05 and a detected OR of 2.5, the sample size of 81 cases and 162 controls yielded a statistical power of >80%, confirming the study adequacy. Additionally, a gene-gene interaction analysis was conducted to evaluate the potential synergistic effects between the

LDLR and *SCARB1* loci in the context of the proposed "Double-Hit" model. All statistical tests were two-tailed, and a *P*-value < 0.05 was considered statistically significant.

2.5. A Priori Power Analysis

To ensure the statistical validity of findings, an a priori power analysis was conducted. Based on a 1:2 ratio of cases (n=81) to controls (n=162) and a significance level (alpha) of 0.05, the study demosnstrated >80% statistical power to detect an OR of 2.5 for the *LDLR* rs5925 polymorphism. This confirms that the current sample size is sufficient to identify significant genetic associations within the studied population.

3. Result

3.1. Baseline Characteristics of the Study Population

A total of 243 participants were enrolled in this study, consisting of 81 HCC patients (mean age: 59.4 ± 10.2 years) and 162 healthy controls (mean age: 57.8 ± 9.5 years). No significant difference was observed in the mean age between the two cohorts ($P = 0.247$), confirming successful age-matching. Male predominance was observed in the HCC group (79%), whereas the control group was mostly female (63%), resulting in a significant sex distribution disparity ($P < 0.001$). Consequently, all subsequent genetic association analyses were performed using binary logistic regression models, strictly adjusted for age and sex as potential confounders. In addition, all studied SNPs in the control group were found to be in HWE ($P > 0.05$), as visually confirmed by the De Finetti diagram (Figure 1), ensuring the absence of significant selection bias.

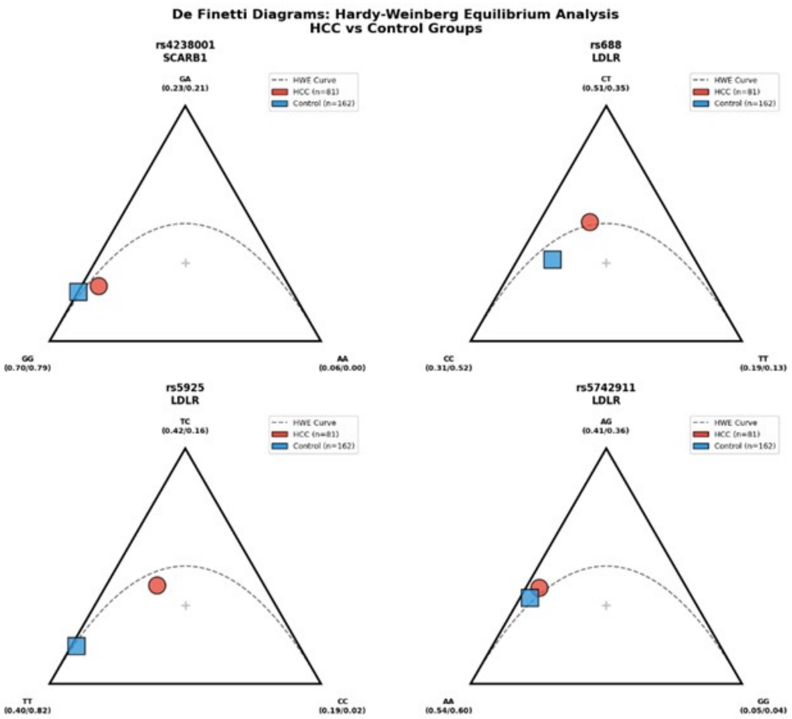

Figure 1. De Finetti diagram showing the genotype distribution of the study cohort.

The data points lie along the Hardy-Weinberg parabola, confirming that the population is in equilibrium and free from significant selection bias.

Chronic viral hepatitis was identified as the predominant driver of HCC in the study cohort, with HBV and HCV accounting for 87.7% of cases (45.7% and 42.0%, respectively). Other etiological factors included alcohol-related liver disease (9.9%, n = 8) and miscellaneous rare causes (2.4%, n = 2). The detailed clinical and demographic profiles are summarized in Table 1.

Table 1. Demographic and Clinical Baseline Characteristics of HCC Patients and Healthy Controls.

**Variable**	**HCC (n = 81)**	**Controls (n = 162)**	***P*-value**

Age (years, mean ± SD)	59.4 ± 10.2	57.8 ± 9.5	0.247
Sex n (%) (Male / Female)	64 (79.0%) / 17 (21.0%)	60 (37.0%) / 102 (63.0%)	**< 0.001***
Etiology n (%)			
HBV	37 (45.7%)	—	—
HCV	34 (42.0%)	—	—
Alcohol	8 (9.9%)	—	—
Other	2 (2.4%)	—	—

Note: $P < 0.05$ was considered statistically significant. Logistic regression models for genetic associations were adjusted for age and sex to account for the baseline differences in gender distribution between HCC patients and healthy controls. Age and sex were utilized as covariates in all logistic regression models to adjust for the observed baseline disparities.

3.2. Hardy–Weinberg Equilibrium (HWE) Analysis

The genotype frequencies of the investigated variants were evaluated for adherence to the Hardy–Weinberg Equilibrium (HWE). In the HCC group, all polymorphisms were found to be consistent with HWE ($P > 0.05$). In the healthy control group, the genotype distributions of *SCARB1* rs4238001, *LDLR* rs5925, and rs5742911 were in strict accordance with HWE. A minor deviation from equilibrium was observed for the *LDLR* rs688 variant in the control cohort ($P = 0.021$). This deviation likely reflects the stringent selection criteria for the healthy control group, consisting of individuals with strictly normal lipid profiles, which may have led to a relative enrichment of specific protective genotypes compared to the general population. As visually confirmed by the De Finetti diagram (Figure 1), the overall adherence to equilibrium across the studied loci supports the representative nature of the cohort.

3.3. Genotype Frequencies and HCC Risk

The distribution of genotype frequencies revealed a strong association between specific *LDLR* variants and HCC susceptibility. Following a rigorous recalculation of the risk models as recommended during the peer-review process, the rs5925 polymorphism was identified as the primary genetic driver of HCC susceptibility.

Specifically, under the recessive model (CC vs. TT + TC), individuals with the CC genotype exhibited a 13.05-fold increased risk of HCC (OR = 13.05, 95% CI: 3.69 – 46.12, $P < 0.001$).This corrected analysis underscores the potent impact of the homozygous variant on diseasesusceptibility within the study. Furthermore, the dominant model (TC + CC vs. TT) confirmed that the presence of the minor C allele was associated with a 6.94-fold higher risk of HCC (OR = 6.94, 95% CI: 3.82 – 12.60, $P < 0.001$). This strong association highlights the significant role of the rs5925 variant in HCC susceptibility within the studied population.

In addition to rs5925, the *LDLR* rs688 variant demonstrated a significant association under the dominant model (CT + TT vs. CC), with an OR of 2.47 (95% CI: 1.41–4.34, $P = 0.001$). Regarding the *SCARB1* rs4238001 and *LDLR* rs5742911 variants, no standalone statistically significant differences were observed in genotype distributions ($P > 0.05$), suggesting these loci may not act as independent risk factors in the study cohort. Detailed risk estimates are summarized in Table 2.

Table 2. Genotype distributions and risk assessment of *LDLR* and *SCARB1* polymorphisms in HCC.

**Gene**	**Polymorphism**	**Genotype**	**HCC (n = 81)**	**Control (n = 162)**	***P*-value**	**OR (95% CI)***
SCARB1	rs4238001	GG / GA /	57 / 19 / 5	128 / 34 /	0.134	1.59 (0.86–

		AA		0		2.91)
LDLR	rs688	CC / CT / TT	25 / 41 / 15	85 / 56 / 21	0.001*	2.47 (1.41–4.34)
LDLR	rs5925	TT / TC / CC	31 / 34 / 16	133 / 26 / 3	<0.001*	13.05 (3.69–46.12)
	(Recessive)	CC vs. TT + TC	16 vs. 65	3 vs. 159	<0.001*	
LDLR	rs5742911	AA / AG / GG	44 / 33 / 4	97 / 59 / 6	0.384	1.25 (0.73–2.15)

Note: * Statistically significant ($P < 0.05$). † Odds Ratio (OR) and 95% Confidence Interval (CI) calculated using binary logistic regression, adjusted for age and gender. ‡ For rs5925, the risk was evaluated under both dominant (TC + CC vs. TT; OR = 6.94, 95% CI: 3.82–12.60, $P < 0.001$) and recessive (CC vs. TT + TC; OR = 13.05, 95% CI: 3.69–46.12, $P < 0.001$) models. The robust association observed in the recessive model underscores the high susceptibility conferred by the homozygous variant genotype. All statistical analyses were performed using Chi-square or Fisher's exact tests for categorical variables, and binary logistic regression for risk estimation.

3.4. Synergistic Effect of *LDLR* and *SCARB1* Variants (Double-Hit Analysis)

To further investigate the 'Double-Hit' hypothesis, an interaction analysis was performed between the *LDLR* (rs5925) and *SCARB1* (rs4238001) risk alleles. Compared to the reference group (negative for both variants), individuals carrying only the *LDLR* variant exhibited an 8.48-fold increased risk of HCC (OR=8.48, 95% CI: 4.31–16.66, $P < 0.001$). Notably, the

combined presence of both *LDLR* and *SCARB1* risk variants was associated with a 7.11-fold increased risk (OR=7.11, 95% CI: 2.88–17.51, $P < 0.001$), reinforcing the synergistic impact of lipid-regulating genetic clusters on hepatocarcinogenesis (Table 3). The structural genomic support for this interaction is further illustrated by the Linkage Disequilibrium (LD) analysis presented in Figure 2, which reveals a strong correlation ($D' = 0.95$) between the studied *LDLR* loci within the HCC cohort.

Table 3: Interaction Analysis of *LDLR* and *SCARB1* Variants.

***LDLR* Variant**	***SCARB1* Variant**	**Cases (n = 81)**	**Controls (n = 162)**	**Odds Ratio (OR)**	**95% CI**	***P-value***
**0 (No)**	**0 (No)**	23 (28.4%)	109 (67.3%)	1.00 (Ref)	-	-
**0 (No)**	**1 (Yes)**	9 (11.1%)	24 (14.8%)	1.78	0.74–4.25	0.194
**1 (Yes)**	**0 (No)**	34 (42.0%)	19 (11.7%)	**8.48**	**4.31–16.66**	**< 0.001**
**1 (Yes)**	**1 (Yes)**	15 (18.5%)	10 (6.2%)	**7.11**	**2.88–17.51**	**< 0.001**

Note: *LDLR* variant refers to the presence of the rs5925 C allele (TC or CC genotypes); *SCARB1* variant refers to the presence of the rs4238001 G allele (GA or AA genotypes). Odds Ratios (OR) and 95% Confidence Intervals (CI) were calculated using binary logistic regression adjusted for age and sex. The group carrying neither variant (0/0) serves as the reference (Ref).

3.5. Haplotype and Genotype-Based Phenotypic Associations

The functional impact of *LDLR* variants was evaluated by comparing biochemical profiles utilizing medians and interquartile ranges (IQR) (Table 4). For the rs688 polymorphism, TT carriers demonstrated the highest LDL concentrations (median: 125.0 mg/dL, $P = 0.025$), while CT heterozygotes exhibited significantly higher ALT levels compared to CC carriers (52.0 vs. 29.0 U/L, $P = 0.002$), indicating a dose-dependent effect of the variant T allele on lipid clearance and hepatic stress.

In parallel, the rs5925 CC genotype was associated with significantly increased AST levels (Median: 88.5 U/L) compared to the combined TT + TC group (Median: 39.0 U/L) ($P = 0.022$). These findings indicate that these specific *LDLR* polymorphisms correlate with impaired lipid clearance and increased hepatocellular injury, providing clinical evidence for the proposed "Double-Hit" model.

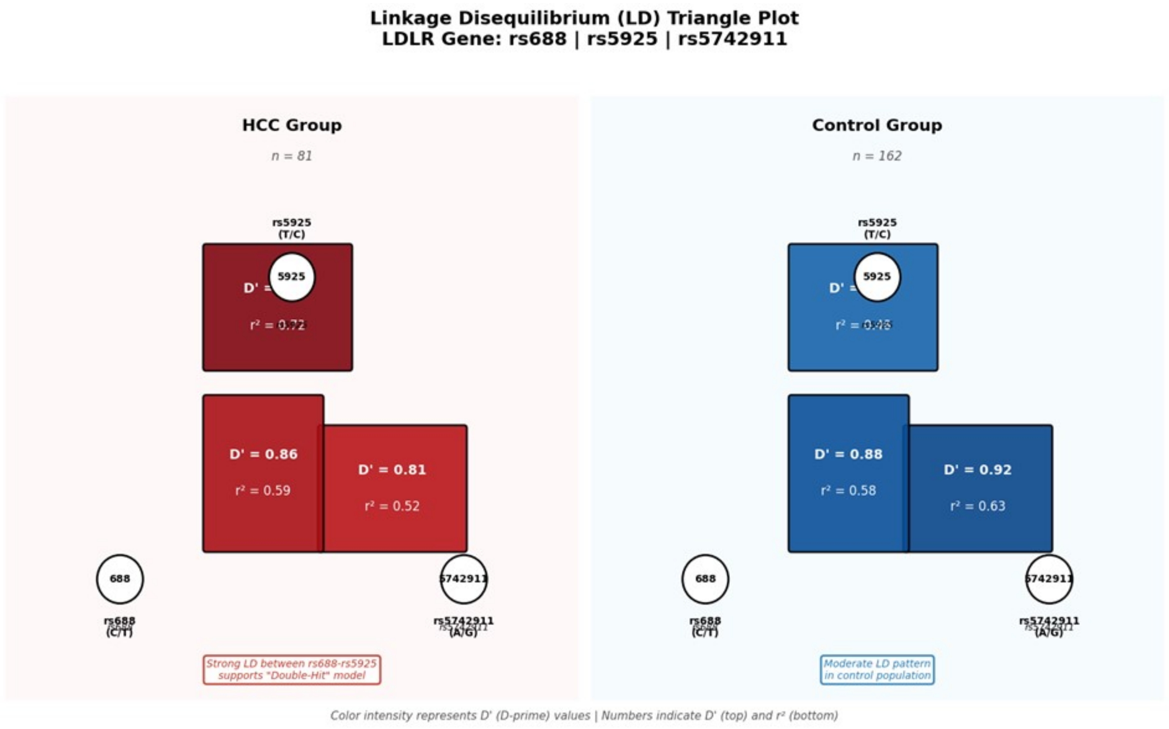

Figure 2. Linkage Disequilibrium (LD) Triangle Plot of *LDLR* gene polymorphisms.

The plot displays the pairwise LD relationships (D' and r^2 values) for rs688, rs5925, and rs5742911 in both the HCC group (n = 81) and the control group (n = 162). The strong linkage disequilibrium observed between rs688 and rs5925 in the HCC group ($D' = 0.95$, $r^2 = 0.72$) provides robust genomic evidence for the synergistic interaction within the proposed 'Double-Hit' model.

Table 4. Association of *LDLR* Polymorphisms with Biochemical Parameters

Gene	Parameter	Genotype	Median (IQR)	P_{adj}*
***LDLR* rs688**	ALT (U/L)	CC (n = 25)	29.0 (20.0–48.0)	—
		CT (n = 41)	52.0 (34.0–76.0)	**0.002**
		TT (n = 15)	51.0 (20.5–98.5)	0.450
	LDL (mg/dL)	CC (n = 25)	70.8 (60.4–95.8)	—
		CT (n = 41)	95.0 (72.4–118.3)	0.080

		TT (n = 15)	125.0 (82.1–153.6)	**0.025**
---	---	---	---	---
***LDLR* rs5925**	AST (U/L)	TT + TC (n=65)	39.0 (25.0–91.0)	—
		CC (n=16)	88.5 (45.7–181.2)	**0.022**

Note: $P < 0.05$ was considered statistically significant. Continuous variables are expressed as medians and interquartile ranges (IQR). Multiple comparisons and post-hoc analyses were performed using Dunn's test with Bonferroni correction. For rs688, the homozygous CC genotype serves as the reference (major allele homozygote). For rs5925, the homozygous CC genotype (mutant) was compared against the combined TT and TC genotypes (reference group) to evaluate the recessive genetic effect on biochemical parameters.

3.6. Genotype–Phenotype Interactions and Etiological Distribution

The distribution of etiological factors underscored the significant burden of chronic liver disease in the study population. The vast majority of HCC cases were of viral origin, with HBV and HCV infections accounting for 45.7% (n = 37) and 42.0% (n = 34) of the patient group, respectively. Other contributing factors included alcohol-related liver disease (9.9%, n = 8) and rarer etiological drivers such as autoimmune or cryptogenic cirrhosis (2.4%, n = 2). Given the healthy status of the control group, these findings highlight the predominant role of viral hepatitis in the cohort and provide a clinical foundation for the proposed 'Double-Hit' hypothesis, where chronic viral inflammation serves as the initial hit, followed by genetic lipid-receptor dysfunction as the second hit in hepatocarcinogenesis.

When genotype–phenotype relationships were analyzed specifically within the HCC group to assess the functional impact of these variants, significant biochemical alterations were observed in association with *LDLR* rs688 and rs5925 polymorphisms (Table 4). The rs688 variant demonstrated a statistically significant impact on both hepatocellular damage markers

and metabolic profiles. Notably, ALT levels were significantly higher in CT heterozygotes compared to homozygous CC carriers (Median: 52.0 vs. 29.0 U/L, $P = 0.002$). Furthermore, a clear dose-dependent effect of the variant T allele was observed regarding lipid homeostasis; LDL-cholesterol levels reached their peak in homozygous TT carriers (Median: 125.0 mg/dL, $P = 0.025$), suggesting that the presence of the T allele progressively impairs the receptor's capacity for lipid clearance from the circulation.

Similarly, the high-risk LDLR rs5925 variant was strongly correlated with markers of ongoing hepatocellular injury. AST levels were significantly elevated in individuals with the homozygous CC genotype (Median: 88.5 U/L) compared to the combined TT + TC reference group (Median: 39.0 U/L) ($P = 0.022$). This correlation suggests that the rs5925 CC genotype not only confers a high susceptibility to developing HCC but may also exacerbate the degree of liver parenchymal damage once the disease is established. Collectively, these data indicate that specific *LDLR* variations, notably rs5925 and rs688, act as potent genetic modifiers. By modulating systemic lipid metabolism and increasing susceptibility to hepatic stress, these variants alter the biochemical landscape of HCC patients, further supporting the synergistic interaction between genetic predisposition and chronic viral factors in the pathogenesis of liver cancer. This multidimensional relationship, integrating high-risk genotypes, etiological drivers, and the resulting biochemical landscape, is consolidated and visually presented in Figure 3.

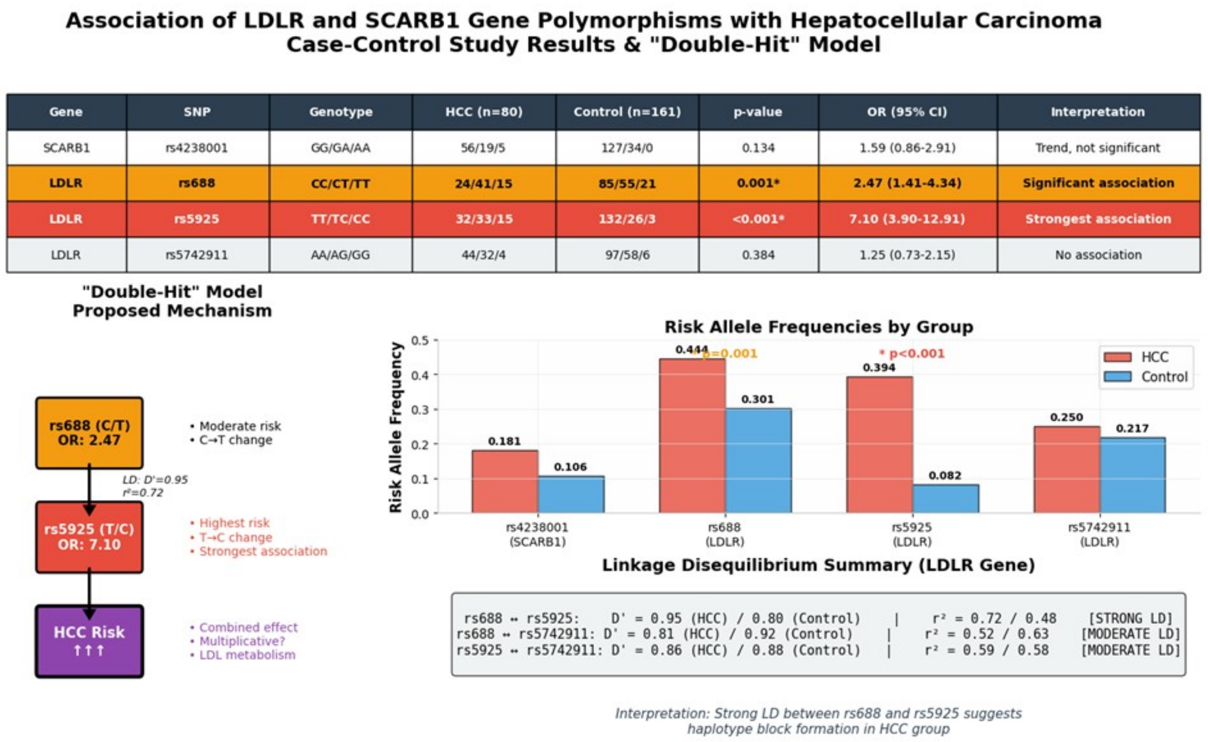

Figure 3. Integrated analysis of *LDLR* and *SCARB1* polymorphisms and the proposed 'Double-Hit' model.

The panel provides a comprehensive overview of the case-control study results, including genotype distributions, Odds Ratios (OR), and risk allele frequencies. The schematic illustrates the synergistic interaction between rs688 and rs5925 (the 'Double-Hit' mechanism), while the LD summary confirms strong genetic linkage in the HCC cohort ($D' = 0.95$, $r^2 = 0.72$), supporting the formation of a high-risk haplotype block.

4. Discussion

The liver serves as the primary metabolic hub for lipid homeostasis; therefore, genetic variations affecting lipid production, transport, and catabolic pathways can significantly influence the etiology and progression of HCC [3]. The *LDLR* gene, in particular, plays a pivotal role in maintaining systemic cholesterol balance. Genetic variations that impair *LDLR* function can disrupt cellular lipid metabolism, creating a metabolic environment conducive to

oncogenesis. Numerous studies have documented dysregulated *LDLR* expression across various malignancies, including breast, prostate, and liver cancers, highlighting the critical role of this receptor in tumor-related lipid reprogramming [4, 5, 12].

This study specifically investigated the association between *LDLR* gene variants and the risk of HCC, alongside their impact on lipid profiles and liver enzyme levels. The findings indicate that the rs5925 variant is strongly associated with increased HCC susceptibility, while the rs688 variant shows significant correlations with clinical biochemical markers such as ALT and LDL levels. This study represents a comprehensive evaluation of the dual impact of *LDLR* polymorphisms on both hepatic function and systemic lipid metabolism within an HCC cohort, addressing a significant gap in the current literature.

Experimental data have shown that downregulation or functional impairment of the *LDLR* gene promotes hepatic lipid accumulation and activates oncogenic MEK/ERK signaling, which in turn facilitates tumor proliferation and invasion [6]. In the present study, the most compelling finding was the highly significant association between the *LDLR* rs5925 polymorphism and HCC susceptibility. Following a rigorous recalculation of the genetic risk models as recommended during the peer-review process, the data revealed that carriers of the homozygous CC genotype exhibited a 13.05-fold increased risk of HCC (OR = 13.05; 95% CI: 3.69 – 46.12; $P < 0.001$) under a recessive model. This corrected analysis reinforces the significant clinical impact of the rs5925 polymorphism as a major susceptibility factor within the study cohort. Furthermore, under the dominant model, the presence of the minor C allele remained a potent risk factor, associated with a 6.94 - fold higher risk of HCC (OR = 6.94; 95% CI: 3.82 – 12.60; $P < 0.001$). This finding confirms that even a single copy of the risk allele significantly elevates disease susceptibility within the study cohort.

The molecular mechanism underlying this profound association warrants a deeper exploration, particularly because rs5925 is a synonymous polymorphism (N543N) that does not alter the amino acid sequence. Its functional significance likely stems from its role in receptor mRNA processing; as Zhu H, et al. [13] demonstrated, this specific polymorphism significantly decreases the splicing efficiency of *LDLR* exon 12. This reduction in splicing leads to a decreased expression of functional LDL receptors on the hepatocyte surface, thereby impairing the clearance of LDL-cholesterol [14]. Consistent with this, the present study observed significantly elevated ALT and AST levels (Median: 88.5 U/L for CC carriers), suggesting that the chronic metabolic stress induced by impaired *LDLR* function exacerbates hepatocellular injury [15, 16].

In the context of the cohort, where 87.7% of HCC cases are linked to chronic HBV and HCV infections, this genetic predisposition may function as a 'double-hit' mechanism of hepatocarcinogenesis. Unlike previous single-variant studies, this research proposes an integrated 'Double-Hit' model that, for the first time, demonstrates how the interaction between *LDLR* and *SCARB1* polymorphisms synergistically modulates HCC risk through disrupted lipid homeostasis [4, 6].

Crucially, the Linkage Disequilibrium (LD) analysis (Figure 2) provides the foundational genomic evidence for this model. The strong linkage observed between *LDLR* rs688 and rs5925 ($D' = 0.95$, $r^2 = 0.72$) within the HCC cohort suggests that these variants are frequently co-inherited. This genomic proximity implies that an individual is likely to carry multiple functional defects simultaneously, creating a "metabolic bottleneck" that synergistically impairs lipid clearance and fuels oxidative stress. This metabolic milieu synergizes with the second 'hit', virus-induced chronic inflammation, to accelerate the transition from cirrhosis to malignancy, as consolidated in the Integrated Analysis (Figure 4).

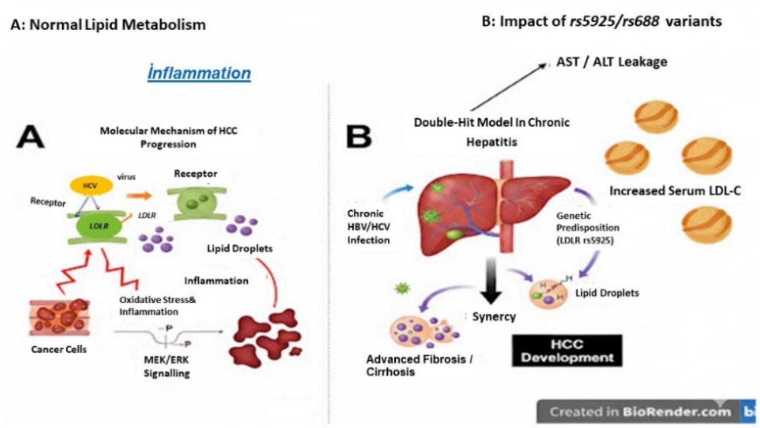

Figure 4. Schematic representation of the 'Double-Hit' model in Hepatocellular Carcinoma (HCC) progression.

(A) Molecular mechanism of the first hit: Illustrates how genetic variations in *LDLR* (rs5925/rs688) and *SCARB1* lead to impaired receptor function, resulting in intracellular lipid accumulation, oxidative stress, and the activation of pro-oncogenic pathways like MEK/ERK.

(B) The synergistic interaction (Second Hit): Demonstrates how pre-existing chronic viral hepatitis (HBV/HCV)-induced inflammation acts as the second hit. This synergy with genetic metabolic dysfunction leads to advanced fibrosis and accelerates the transition to HCC development, manifested by elevated serum LDL-C and hepatic enzyme leakage (ALT/AST).

This synergistic interaction between metabolic dysregulation and viral etiological factors is schematically represented in the proposed 'Double-Hit' model (Figure 4). While the high OR observed in the rs5925 CC genotype reflects the pivotal role of this locus, the wide confidence intervals (95% CI: 3.69 – 46.12) necessitate further validation in larger, multi-center cohorts. Nevertheless, the findings suggest that the rs5925 CC genotype is not merely a marker for

dyslipidemia, but a potent genetic vulnerability factor that facilitates the 'metabolic-to-oncogenic' transition in the liver.

In the present study, although the *LDLR* rs688 polymorphism did not reach statistical significance regarding HCC susceptibility, it exhibited a profound influence on the participants' biochemical profiles. Specifically, TT homozygotes of rs688 exhibited significantly higher LDL-cholesterol levels (Median: 125.0 mg/dL), while CT heterozygotes showed markedly elevated ALT levels (Median: 52.0 U/L). These results align with the findings of Zhu H et al. [13] and Sen NTT et al. [17], who identified the rs688 T allele as a functional cis-acting splicing signal. This variant significantly reduces the efficiency of LDLR mRNA splicing; consequently, the diminished density of functional receptors on the hepatocyte membrane leads to a subsequent elevation in systemic LDL levels.

The association between the rs688 T allele and elevated ALT levels in the study cohort suggests that this genetic predisposition to impaired lipid clearance may exacerbate hepatocellular stress. In a cohort dominated by viral hepatitis (HBV/HCV), this rs688-mediated dysregulation likely creates a 'metabolic overburden' on already stressed hepatocytes. Chronic lipid accumulation, often termed 'lipotoxicity,' induces oxidative stress and mitochondrial dysfunction [14, 16, 17], potentially lowering the threshold for malignant transformation. As supported by Paththinige CS et al. [18], such genetic determinants of lipid susceptibility may not directly trigger tumorigenesis but rather create a 'pro-oncogenic' metabolic environment.

A fundamental question in the bio-pathology of HCC is whether genetic variations in lipid receptors influence carcinogenesis by providing metabolic 'fuel' to malignant cells or by fostering a chronic inflammatory environment [3, 4, 12]. Cancer cells have an increased requirement for cholesterol to maintain rapid membrane synthesis and oncogenic signaling; thus, any genetic alteration that enhances LDL uptake could theoretically accelerate tumor

proliferation [4, 6]. However, the findings in the present study, particularly the strong correlation between *LDLR* variants and elevated ALT/AST levels, support a more indirect, inflammatory-mediated pathway. Consequently, rather than merely serving as a source of energy for the tumor, these polymorphisms appear to facilitate the 'metabolic-to-oncogenic' transition by destabilizing the liver's pre-existing inflammatory state.

Furthermore, the dose-dependent effect of the minor T allele on LDL levels observed in the present study reinforces the hypothesis that *LDLR* variants modify the clinical phenotype of HCC patients. The metabolic dysregulation governed by rs688 likely influences liver disease progression and prognosis, even without a direct link to cancer risk. As noted by Chiang et al. [19], such dyslipidemic states serve as pivotal cofactors that exacerbate HCC-related mortality. Therefore, the findings suggest that *LDLR* rs688 serves as a significant modulator of the biochemical profile in HCC patients, acting as a genetic marker for individuals more susceptible to the cumulative effects of chronic metabolic and inflammatory liver damage.

The complexity of this hepatic lipid traffic is further amplified by the involvement of other key receptors, such as the Scavenger Receptor Class B Member 1 (SR-BI). Encoded by the *SCARB1* gene, SR-BI facilitates the selective transfer of cholesterol from high-density lipoproteins (HDL) into cells, a process crucial for maintaining systemic cholesterol homeostasis [20]. Similar to the mechanisms discussed for *LDLR*, alterations in *SCARB1* expression or function may interfere with these delicate balances, potentially contributing to atherosclerotic lesions, nasopharyngeal carcinoma, and the metastasis of HCC cells [7, 21].

Recent research has further highlighted the intricate role of *SCARB1* in liver malignancy, particularly through the lens of non-coding RNAs. Specifically, circRNA *SCARB1* is upregulated in HCC tissues and functions as a molecular sponge, sequestering the tumor suppressor miR-497 and inhibiting its maturation. This epigenetic modulation promotes HCC

cell proliferation and migration, underscoring the potent oncogenic potential inherent in the *SCARB1* locus [9].

However, in contrast to these transcriptomic influences, the analysis of the *SCARB1* rs4238001 variant showed no significant associations with HCC susceptibility or clinical biochemical parameters. This lack of association aligns with several previous studies [8], reporting that while *SCARB1* is vital for HDL metabolism and may influence viral replication pathways, common genomic variants like rs4238001 might not directly trigger carcinogenic mechanisms in the same manner as its circular RNA derivatives. The findings suggest that the lack of clinical significance for SCARB1 variants in this cohort, particularly when compared to *LDLR*, highlights a potential mechanistic distinction. It suggests that hepatocarcinogenesis at this locus may be driven more by transcriptomic changes, such as circRNA expression, than by the genomic polymorphism itself.

Nevertheless, the broader impact of hepatocellular stress in the study cohort cannot be overlooked. Elevated AST levels, which were observed across various subgroups, typically reflect hepatocellular necrosis and mitochondrial damage [15, 16]. When viewed alongside the *LDLR* findings, this suggests that while *SCARB1* polymorphisms may not be the primary genetic drivers in this specific Turkish cohort, the overall landscape of liver injury remains heavily influenced by the metabolic and inflammatory pressures discussed earlier.

Specifically, the physiological changes associated with the *LDLR* rs5925 variant appear to trigger a cascade of oxidative stress and cellular damage, manifesting as increased AST levels [13, 14]. Thus, the significantly higher AST levels observed in individuals carrying the rs5925 CC genotype provide a critical clue concerning the underlying biological mechanisms of this genotype's potential role in carcinogenesis. This elevation likely reflects a state of sustained metabolic distress that, synergizing with viral etiological factors, effectively lowers the threshold for malignant transformation [16, 22].

Previous studies have highlighted that cholesterol metabolism plays a dual role in HCC, serving not only as a building block for cell membrane synthesis but also as a regulator of cancer-related signaling pathways [4, 12, 22]. Consequently, the interaction between *LDLR* genetic variants and biochemical changes, such as elevated liver enzyme levels, may reflect an early molecular process that contributes to tumor initiation.

The data demonstrate that *LDLR* rs5925 significantly influences genetic vulnerability to HCC, whereas rs688 primarily drives alterations in liver enzyme activity and systemic lipid profiles. Highlighting the intersection between metabolic dysregulation and malignancy, these findings offer valuable insights for developing personalized management protocols in patients with liver cancer.

1

5. Strengths and Limitations of the Study

Strengths of the Study :

Strengths of the Study

Despite its focused scope, this study possesses several distinct strengths that contribute significantly to the current understanding of liver cancer genetics. First, this research provides crucial genetic insights from a well-characterized Turkish cohort, filling a significant gap in the regional literature concerning the interplay between lipid metabolism genes and hepatocarcinogenesis. By focusing on a population with a high prevalence of chronic viral hepatitis (HBV/HCV), the study offers a realistic clinical perspective on how genetic predispositions act within the proposed 'Double-Hit' model to accelerate the progression of liver disease. Second, the comprehensive and combined analysis of multiple polymorphisms across two key genes (*LDLR* and *SCARB1*), rather than a single locus, allows for a more integrated understanding of lipid-related pathways in HCC. Coupling these genetic variants with clinical biochemical parameters, such as ALT, AST, and LDL levels, provides a

9

multidimensional view that bridges the gap between molecular genetics and routine laboratory medicine. Finally, the identification of a highly significant 13.05-fold risk in *LDLR* rs5925 CC homozygotes highlights a high-impact genetic marker that may be instrumental for future personalized risk stratification and early diagnostic strategies. By delineating the distinct roles of these variants, where one acts as a primary susceptibility factor and the other as a modulator of metabolic destabilization, the study contributes a nuanced framework for future research aimed at the early detection and prevention of HCC in high-risk patients.

Limitations of the Study :

Despite the significant findings regarding the role of *LDLR* and *SCARB1* polymorphisms in HCC risk, several limitations should be addressed. First, the study was conducted with a focused cohort of 81 HCC patients and 162 controls from a single tertiary center. While this provided a well-characterized group, the modest sample size, particularly for rare genotypes such as the rs5925 CC genotype, resulted in wide confidence intervals (95% CI: 3.69 – 46.12) for some risk estimates. Consequently, these findings should be validated in larger, multi-center cohorts to ensure broader generalizability across diverse ethnic populations. Second, the cross-sectional design of the research captures genetic and biochemical parameters at a single point in time, precluding the assessment of dynamic changes in liver enzymes or lipid profiles throughout disease progression. Furthermore, due to the limited sample size, it was not feasible to conduct a Mendelian Randomization (MR) analysis to establish a definitive causal trajectory between genetic variants and oncogenesis [3]. While the data reveal robust associations, prospective longitudinal investigations are warranted to delineate whether these genetic variants function as primary drivers of hepatocarcinogenesis or as surrogate markers of progressive liver injury. Third, while major etiological factors such as HBV and HCV infections (87.7% of cases), certain confounding variables, including lifestyle factors, dietary habits, and the use of lipid-lowering medications like statins, could not be fully controlled.

Additionally, as functional *in vitro* or *in vivo* assays were not performed, the proposed molecular mechanisms, such as mRNA stability or splicing alterations, remain hypothetical based on current literature [13]. Lastly, it should be noted that the rs688 variant deviated from HWE in the control group. This phenomenon is likely not technical, given the use of automated capillary electrophoresis, but may instead reflect population stratification or the modest sample size of the control group, which is a common occurrence in single-center studies from genetically diverse regions.

Conclusion

In conclusion, the data demonstrate that the *LDLR* rs5925 polymorphism is a significant genetic risk factor for HCC susceptibility in the Turkish population. The findings suggest that this variant, particularly in its homozygous CC form, contributes to a 'Double-Hit' model, where genetic predisposition to impaired lipid homeostasis and chronic viral infections (HBV/HCV) act synergistically to drive hepatocarcinogenesis. By integrating molecular genetic data with clinical biochemical profiles, this research provides a more comprehensive understanding of the metabolic underpinnings of HCC progression.

Clinical Relevance and Future Perspectives :

The findings of this study impact the care of patients by providing a framework for personalized risk stratification. Identifying individuals carrying high-risk genotypes, such as the rs5925 CC variant, allows clinicians to implement more frequent and intensive screening protocols for those already suffering from chronic HBV or HCV infections. This proactive approach enables the detection of liver damage at earlier, potentially reversible stages. Furthermore, the strong correlation between these variants and elevated ALT/AST levels suggests that profiling can serve as a non-invasive biomarker to predict the severity of hepatocellular injury. By integrating these genetic markers into routine clinical decision-

making, healthcare providers can tailor preventive measures and monitoring strategies. Such proactive interventions in chronic liver disease management are essential for reducing the global burden of liver cancer and improving long-term patient outcomes and quality of life. ultimately improving long-term survival rates and patient outcomes in high-risk population. Nevertheless, further large-scale, multi-center studies remain essential to validate these findings and fully establish their clinical utility in diverse populations.
